# Noxa inhibits oncogenesis through ZNF519 in gastric cancer and is suppressed by hsa-miR-200b-3p

**DOI:** 10.1038/s41598-024-57099-7

**Published:** 2024-03-19

**Authors:** Jin Shi, Fan Ding, Dezhu Dai, Xudong Song, Xu Wu, Dongsheng Yan, Xiao Han, Guoquan Tao, Weijie Dai

**Affiliations:** 1 Department of Gastrointestinal Surgery, The Affiliated Huaian No. 1 People’s Hospital of Nanjing Medical University, Huai’an, Jiangsu 223300 People’s Republic of China; 2 Department of Vascular, Huaian Hospital Affiliated to Xuzhou Medical University, Huai’an, Jiangsu 223300 People’s Republic of China; 3 Department of Gastroenterology, The Affiliated Huaian No. 1 People’s Hospital of Nanjing Medical University, Huai’an, Jiangsu 223300 People’s Republic of China

**Keywords:** Gastric cancer, Noxa, Hsa-miR-200b-3p, ZNF519, Prognosis, Proliferation, Migration, Invasion, Cell growth, Cell proliferation, Cancer prevention, Cancer therapy, Gastrointestinal cancer, Cancer

## Abstract

While Phorbol-12-myristate-13-acetate-induced protein 1 (Noxa/PMAIP1) assumes a pivotal role in numerous tumors, its clinical implications and underlying mechanisms of gastric cancer (GC) are yet enigmatic. In this investigation, our primary objective was to scrutinize the clinical relevance and potential mechanisms of Noxa in gastric cancer. Immunohistochemical analysis was conducted on tissue microarrays comprising samples from a meticulously characterized cohort of 84 gastric cancer patients, accompanied by follow-up data, to assess the expression of Noxa. Additionally, Noxa expression levels in gastric cancer clinical samples and cell lines were measured through quantitative real-time polymerase chain reaction (qRT-PCR) and Western blot analysis. The effect of Noxa expression on the prognosis of patients with gastric cancer was evaluated using Kaplan–Meier survival. Further insight into the role of Noxa in driving gastric cancer progression was gained through an array of experimental techniques, including cell viability assays (CCK8), plate cloning assays, transwell assays, scratch assays, and real-time cell analysis (RTCA). Potential upstream microRNAs (miRNAs) that might modulate Noxa were identified through rigorous bioinformatics analysis, substantiated by luciferase reporter assays and Western blot experiments. Additionally, we utilized RNA sequencing, qRT-PCR, and Western blot to identify proteins binding to Noxa and potential downstream target. Finally, we utilized BALB/c nude mice to explore the role of Noxa in vivo. Our investigation unveiled a marked downregulation of Noxa expression in gastric cancer and underscored its significance as a pivotal prognostic factor influencing overall survival (OS). Noxa overexpression exerted a substantial inhibitory effect on the proliferation, migration and invasion of GC cells. Bioinformatic analysis and dual luciferase reporter assays unveiled the capacity of hsa-miR-200b-3p to interact with the 3′-UTR of Noxa mRNA, thereby orchestrating a downregulation of Noxa expression in vitro, consequently promoting tumor progression in GC. Our transcriptome analysis, coupled with mechanistic validation, elucidated a role for Noxa in modulating the expression of ZNF519 in the Mitophagy-animal pathway. The depletion of ZNF519 effectively reversed the oncogenic attributes induced by Noxa. Upregulation of Noxa expression suppressed the tumorigenesis of GC in vivo. The current investigation sheds light on the pivotal role of the hsa-miR-200b-3p/Noxa/ZNF519 axis in elucidating the pathogenesis of gastric cancer, offering a promising avenue for targeted therapeutic interventions in the management of this challenging malignancy.

## Introduction

Gastric cancer (GC), a common tumour of the gastrointestinal tract, is prevalent worldwide^1^. Over the past few decades, gastric cancer incidence has considerably dropped, but it remains a serious disease that causes many deaths worldwide^2^. Epidemiological research has identified risk factors for stomach cancer, such as Helicobacter pylori infection, advanced age, excessive salt consumption, and a diet deficient in fruits and vegetables^3,4^. The early stages of gastric cancer can manifest as asymptomatic or present with atypical symptoms, often resulting in delayed diagnosis and a substantial portion of patients being identified at an advanced disease stage, posing significant therapeutic challenges^5,6^. Emerging studies have underscored the potential of molecular targeted therapy in the context of gastric cancer, offering promising prospects for treatment^7,8^. Molecularly targeted drugs exert their therapeutic efficacy by selectively acting on specific molecules^9^. Consequently, the quest for novel biomarkers associated with gastric cancer and the development of new drug targets assume paramount importance for the prognosis and treatment of this malignancy.

B-cell lymphoma/leukaemia-2 (Bcl-2) proteins, instrumental in the intrinsic apoptotic pathway, occupy a pivotal role in maintaining the delicate equilibrium between cancer cell survival and apoptosis^10,11^. These proteins can be categorized into three subfamilies: anti-apoptotic, BH3-only, and pro-apoptotic proteins^12^. Among them, Noxa stands out as a crucial BH3-only protein with a significant role in apoptosis^13,14^. Originally identified as one of the p53-inducible genes, Noxa, a member of the Bcl-2 family, assumes a pivotal role in governing intrinsic apoptotic pathways. It exerts its influence on the delicate balance between cancer cell survival and apoptosis through interactions with other Bcl-2 family members^15,16^. Noxa's multifaceted contributions extend to various types of malignancies, where it influences cancer initiation and progression by modulating molecular and cellular biological processes.

Noxa's dual role in apoptosis induction and DNA damage repair renders it a pivotal player in cancer progression and an attractive therapeutic target. Extensive research has demonstrated Noxa's significance in rheumatic diseases, malignant tumors, and anticancer drug responses, including its involvement in conditions such as rheumatoid arthritis^17^, colorectal cancer^18^, lung cancer^19^, and sensitization to anticancer agents^20^. However, investigations into Noxa's involvement in gastric cancer pathogenesis and its interactions within the gastric cancer tumor microenvironment remain relatively limited. Therefore, comprehensive analysis of Noxa's mechanistic roles in gastric cancer and its interplay within the gastric cancer tumor microenvironment represents an essential avenue for future research, shedding further light on Noxa as a promising therapeutic target for gastric cancer.

Our research focused on the expression of Noxa, its role in gastric cancer and the molecular mechanisms that regulate Noxa both upstream and downstream. Our main objective was to aid in the creation of more efficient therapeutic strategies for the treatment of GC.

## Materials and methods

### Gastric clinical tissue and GC cell lines

The training cohort human GC microarray for Noxa staining was bought from Shanghai Outdo Biotech in China. This training cohort comprises a total of 180 samples(90 pairs), with 90 tumor samples and 90 normal adjacent tissue samples, exclusively utilized for the immunohistochemistry (IHC) staining of Noxa. Six cases were lost to follow-up, resulting in a total of 84 pairs of samples included in the analysis. In addition to the training cohort acquired from Shanghai Outdo Biotech, we also collected tissue samples from 48 patients with gastric cancer, including tumors and adjacent normal tissues, from the Affiliated Huaian No.1 People’s Hospital of Nanjing Medical University. These samples were utilized to perform qRT-PCR analysis to evaluate the expression of Noxa, ZNF519, miR-200b-3p and miR-200c-3p. All 48 patients had undergone radical surgery between 2019 and 2023. All patients included in the study willingly participated and provided their informed consent by signing the respective consent form. The study was approved by the Ethics Committee of Huai'an First People's Hospital affiliated with Nanjing Medical University.

Human gastric cancer cell lines, including AGS, HCG-27, and MKN-28, as well as the normal gastric cell line GES-1 and HEK293T, were procured from Shanghai Zhong Qiao Xin Zhou Biotechnology Co.,Ltd. The growth media used for these cell lines was DMEM or RPMI1640 with 10% fetal bovine serum and 1% penicillin–streptomycin (PS). The incubation conditions were set at 37 °C with a 5% CO_2_ atmosphere.

### Plasmid construction and cell transfection

The cells underwent transient transfection procedures using plasmids, siRNAs, hsa-miR-200b-3p mimics, hsa-miR-200b-3p inhibitors, and other specific siRNAs (obtained from GenePharma, Shanghai, China). These transfections were performed according to the manufacturer's instructions using the Lipofectamine 3000 Kit (Thermo Fisher, CA, USA). Transfection efficiency was assessed by harvesting the cells 48 h post-transfection. Detailed sequences are provided in Table S1.

### RNA isolation and qRT-PCR

The TRIzol reagent (Thermo Fisher, CA, USA) was used to extract total RNA from tissues or cells. Subsequently, cDNA was synthesized through a reverse transcription kit. qRT-PCR assays were conducted utilizing a universal SYBR Green Master Mix (Vazyme, Nanjing, China), strictly adhering to the manufacturer's protocols. Regarding miRNAs, miRNA expression levels were quantified using the miRNA RT/qPCR Detection kit (Proteinbio, Nanjing, China) under standard temperature conditions. The calculation of relative mRNA expression levels was conducted utilizing the threshold cycling (2^−∆∆Ct^) method^21^. Relative mRNA expression was normalized to GAPDH, while RNU6B served as an internal normalization standard for miRNA. All the primers used are detailed in Table S2.

### Immunoblot analysis

Proteins were extracted from both treated and untreated cells or tissue samples using RIPA buffer (Thermo Fisher, CA, USA). Bicinchoninic acid (BCA) method (Biosharp, Shanghai, China) was used to measure and estimate the protein concentrations. Subsequently, the protein lysates were separated by SDS-PAGE and subsequently transferred to PVDF membranes (Millipore, Bedford, MA). The membranes were incubated with the primary antibody for an overnight period at 4 °C after a 2-h block at room temperature with 5% milk. Afterward, they were subjected to a 1-h incubation at room temperature with the secondary antibody, and the protein bands were visualized with the ECL chromogenic substrate. Used antibodies were Noxa (1:500 dilution; #A9801, ABclonal), ZNF519 (1:1000 dilution; #A9801, Origene). GAPDH (1:5000 dilution; #GTX100118, GeneTex) was utilized as the internal control.

### IHC analysis

Gastric cancer (GC) tissues were initially fixed using 10% formalin and subsequently embedded in paraffin to create tissue microarrays. These tissue sections were then subjected to specific primary antibody treatments. After antigen retrieval, the sections were blocked for 2 h at room temperature with 1% BSA (Thermo Fisher, CA, USA) in TBST buffer. Subsequently, they were incubated with the primary antibodies overnight at 4 °C. Following three washes, the sections were exposed to an HRP polymer-conjugated secondary antibody (Thermo Fisher, CA, USA) at room temperature. Subsequent to this step, the sections were stained using the 3,3-diaminodbenzidine substrate and haematoxylin. Quantitative analysis was performed using StrataQuest software (version 7.1.129, TissueGnostics GmbH, Vienna, Austria).

### Cell proliferation

Gastric cancer cells, following transient transfection, were seeded into 96-well plates at a density of 3000 cells per well. Subsequently, 10 µL of CCK8 solution was introduced into each well, and the absorbance at 450 nm was measured using a microplate reader (Tecan, Switzerland). For colony formation assays, 500 cells were plated in 6-well plates and allowed to grow for 10–14 days. Following this period, the cells were fixed with methanol and stained with 0.1% crystal violet. The number of colonies formed was imaged and analyzed. For real-time cellular analysis, cell viability is monitored using RTCA (Agilent, USA) by following the manufacturer's instructions. Highly sensitive biosensing technology uses a patented microplate with microgold electrodes embedded in the bottom of the well plate, enabling non-invasive detection of cell behavior. The xCELLigence software records the electrical values and translates them to cell indices when cells affix to the E-plate surface and change the electrical impedance of the entire array. First, 50 μL culture media were added in order to measure the background value. After that, seed the cells onto E-plates after combining them with 50 μL of culture fluid. RTCA 2.0 software was used to capture and export data, and Microsoft Excel and GraphPad Prism were used for analysis^22^.

### Migration and matrigel invasion assay

A total of 10,000 cells were suspended in 200 μL of serum-free media and placed in the upper chamber for the transwell assay. In the lower chambers, 600 μL of media containing 10% FBS was added. After 48 h of incubation, cells on the upper surface of the inserts were gently removed using a cotton swab, while the cells on the underside of the membrane were fixed and stained with 0.1% crystal violet (#DZ0053, Leagene Biotechnology, Beijing, China). For the Matrigel invasion assay, a Matrigel-coated Transwell insert was employed, following the same protocol as described above^23^.

### Cell scratch assay

Transfect cells into a 6-well plate. After the cells have filled the wells, mark the back of the 6-well plate with a marker pen, and then draw a straight line longitudinally in the center with the tip of a 20 μL pipette held against a straightedge. Following three washes with PBS, the images were taken under the inverted microscope. The scratch distance was imaged and analysed at 48 h.

### Dual luciferase reporter assay

Luciferase reporter assays were performed in the HEK293 cell line. In accordance with the manufacturer's instructions, cells were cotransfected with miR-200b-3p precursor or miR negative control and pmirGLO-3′UTR vector using EndoFectin™ Max Transfection Reagent (#EF013, GeneCopoeia, USA). Luciferase activity was assayed using a Dual-Luciferase^®^ Reporter Assay System (#E1910,Promega, USA) according to the manufacturer's protocol. The absorbance readings for both firefly and Renilla luciferase activities were quantified using a microtiter plate photometer (Promega, USA).

### RNA sequencing

Genome-wide transcriptional sequencing was undertaken by Allwegene Tech. (Beijing, China). Transcriptome sequencing was carried out to discern mRNA transcripts exhibiting differential expression between P-Noxa AGS cells and negative control (NC) AGS cells. The processes of RNA preparation and the subsequent preparation of transcriptional sequencing libraries strictly followed the manufacturer's provided instructions. The library preparations were sequenced on an Illumina Novaseq 6000 platform by the Beijing Allwegene Technology Company Limited (Beijing, China) and the library was constructed using Illumina (Illumina, San Diego, USA) kits.

### Bioinformatics analysis

We employed various online tools and databases, including starbase (https://starbase.sysu.edu.cn/)^24^, miRanda (http://www.microrna.org/microrna/home.do)^25^, miRDB (https://mirdb.org/)^26^, TarBase 7.0 (http://microrna.gr/tarbase/)^27^, and miRtarBase (http://mirtarbase.mbc.nctu.edu.tw/php/index.php)^28^, to predict potential target miRNAs for Noxa. Subsequently, we analyzed and filtered the results using TarBase 7.0. We also utilized TargetScan (http://www.targetscan.org/vert)^29^ and miRDB (https://mirdb.org/) to predict potential binding sites between the identified target miRNAs and Noxa.

### In vivo xenograft experiments

Four-week-old BALB/c nude mice were purchased from Nanjing Cavens Biotechnology Co., Ltd. (Nanjing, China). AGS cells (1 × 10^7^) transfected with plasmids containing P-Noxa or NC were injected subcutaneously into the axilla of mice. After a span of 4 weeks, the mice were sacrificed, and the tumors were excised. The study adheres to the relevant guidelines and regulations. The research aligns with the Animal Research: Reporting In Vivo Experiments (ARRIVE) Guidelines.

### Carbon dioxide inhalation procedure

Mice were ethically euthanized through carbon dioxide inhalation, following meticulously approved standard operating procedures sanctioned by the IACUC and veterinary authorities. Mice were positioned within a standardized mouse cage, measuring 11 inches in length, 6.5 inches in width, and 5 inches in depth. We then infused carbon dioxide into the box at a rate that replaces 20% of the euthanasia box's volume per minute for 5 min of exposure. We observed the animals for dilated pupils and any signs of respiration, as well as for response to stimuli such as toe pinching. An additional observation period of 2 to 3 min ensued, ensuring a thorough determination of the cessation of vital signs, confirming the humane and ethically sound completion of the euthanasia procedure.

### Statistical analysis

GraphPad Prism Software (version 8.0) was used for statistical analysis. Survival analysis was estimated by Kaplan–Meier analysis. Each assay was conducted with a minimum of three independent replicates. Comparisons between the two groups of quantitative data were assessed using the Student's t-test, and statistical significance was determined at p < 0.05.

### Ethics approval and consent

The research was conducted in compliance with the ethical approval granted by the Animal Care and Use Committee of The Affiliated Huaian No.1 People’s Hospital of Nanjing Medical University^30^. All experiments were performed in accordance with relevant guidelines and regulations.

### Informed consent

All authors give their consent to publish this manuscript.

## Results

### Low expression of Noxa is observed in gastric cancer patients and associated with an unfavorable prognosis

**Figure 1:**
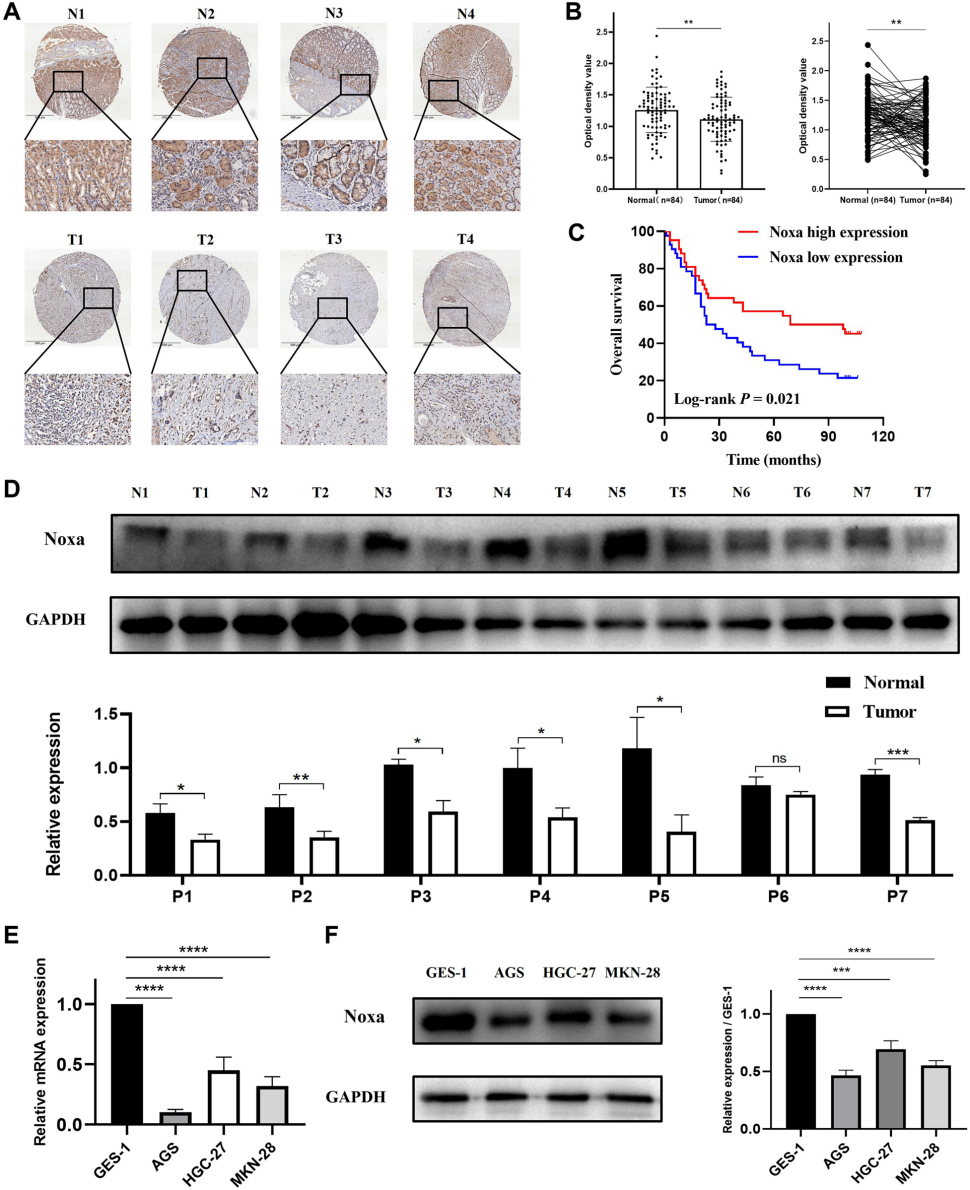
Noxa exhibits low expression levels in gastric cancer and is associated with a poor prognosis. (**A**) Immunohistochemical (IHC) staining of Noxa in tissue microarrays. (**B**) Mean optical density values derived from the analysis of 84 paired sets of gastric cancer tissues and adjacent paracancerous tissues. (**C**) Kaplan–Meier depiction of the OS for the two patient groups with high (n = 42) or low (n = 42) expression of Noxa in GC tissues. (**D**) Immunoblotting experiments examining Noxa expression in 7 paired tissue samples. (**E**,**F**) qRT-PCR and Western blot analysis depicting Noxa gene expression levels in the normal gastric epithelial cell line GES-1 and gastric cancer cell lines AGS, HGC-27, and MKN-28.*P < 0.05; **P < 0.01; ***P < 0.001; ****P < 0.001.

To delineate the expression of Noxa in gastric cancer, we commenced our investigation by conducting immunohistochemical staining on microarrays comprising 84 pairs of gastric cancer tissues and their corresponding adjacent paracancerous tissues. Notably, Noxa expression levels in tumor tissues were discovered to be considerably lower than those in adjacent paracancerous tissues (Fig. 1A). Further quantification via mean optical density analysis corroborated these findings, revealing a substantial downregulation of Noxa expression in GC tissues relative to adjacent paracancerous tissues (*P* = 0.0082) (as illustrated in Fig. 1B). Additionally, our RT-qPCR results demonstrated a reduced expression of Noxa in tumor tissues when contrasted with normal tissues (as illustrated in Fig. S1). Moreover, our Kaplan–Meier analysis unveiled a noteworthy association between low Noxa expression and poor overall survival (OS) among patients (*P* = 0.021) (Fig. 1C). To substantiate this observation, we conducted Western blotting to assess Noxa protein levels in seven randomly selected gastric cancer samples along with their paired normal tissue counterparts. Remarkably, the results indicated a significant reduction in Noxa protein levels in six out of the seven gastric cancer samples when compared to adjacent normal tissues (Fig. 1D). Furthermore, we used qRT-PCR and Western blotting methods to examine Noxa expression in both normal gastric epithelium and gastric cancer cell lines. These investigations revealed that, in contrast to the normal gastric epithelial cell line GES-1, Noxa expression was notably diminished in the three gastric cancer cell lines (AGS, HGC-27, and MKN-28) (Fig. 1E,F). Subsequently, we embarked on an insightful analysis of the relationship between Noxa expression and clinicopathological factors (Table 1). Our findings underscored a strong association between low Noxa expression and advanced T stage (T3-4) as well as the presence of distant metastasis. In conclusion, low Noxa expression was link to a worse prognosis in GC patients.

**Table 1:** Association between Noxa expression and clinicopathological factors.

Characteristic	Total	NOXA expression		*P* value
		Low	High	
Age				
< 60	28	12	16	0.314
≥ 60	55	30	25	
Gender				
Female	30	19	11	0.069
Male	54	23	31	
Pathologic stage				
I–II	38	18	20	0.661
III–IV	46	24	22	
T stage				
T1-2	12	2	10	**0.012**
T3-4	70	39	31	
N stage				
N0	19	8	11	0.399
N+	64	34	30	
M stage				
M0	74	34	40	**0.043**
M1	10	8	2	
Tumor length				
< 5 cm	39	17	22	0.370
≥ 5 cm	43	23	20	
P53 expression				
< 50%	43	26	17	0.093
≥ 50%	34	14	20	

Significant values are in bold.

### Enhanced Noxa expression suppresses the progression of GC cells 

**Figure 2:**
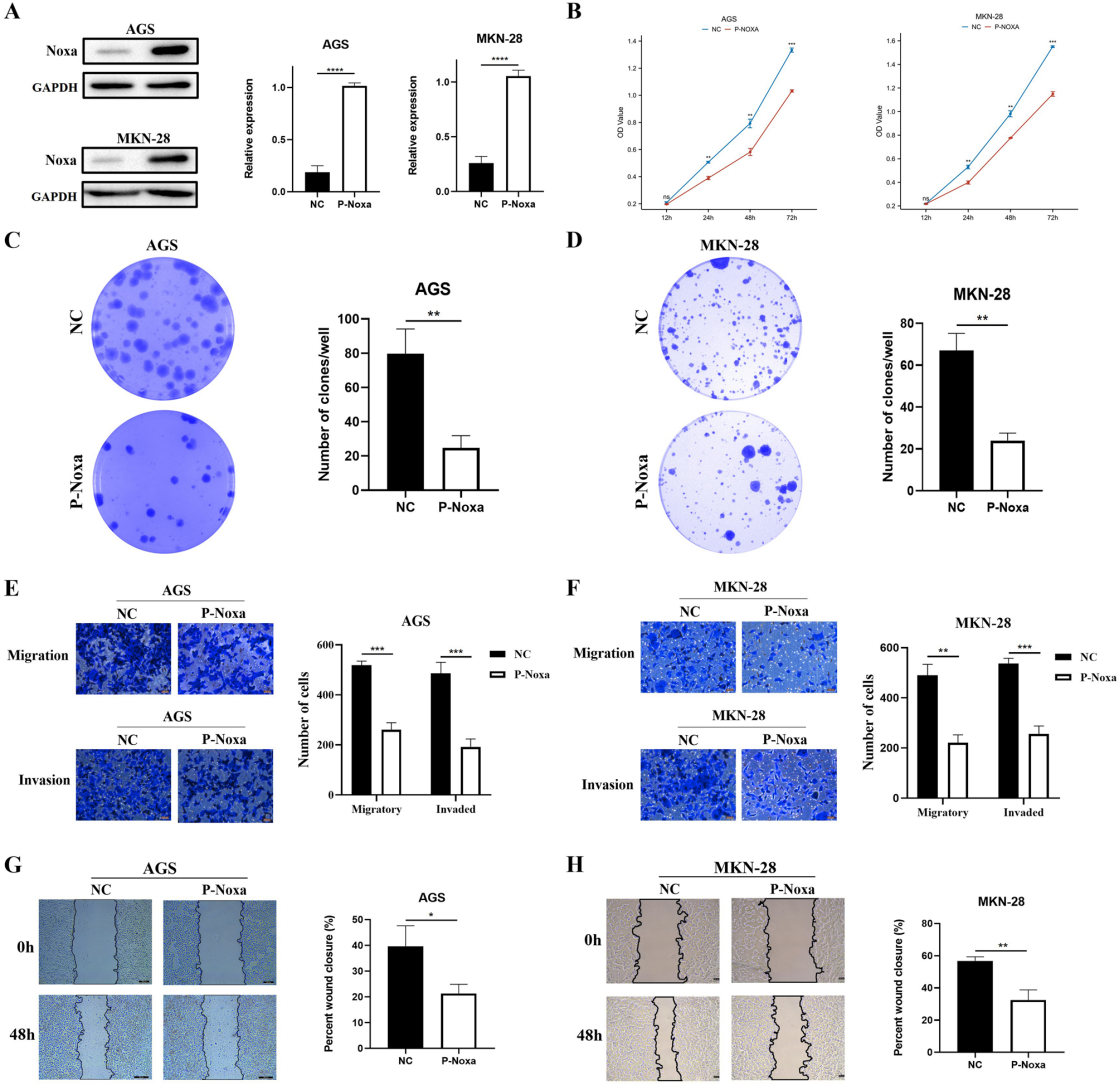
Noxa inhibits GC cell progression. (**A**) Validation of Noxa overexpression in AGS and MKN-28 cells through Western blot analysis. (**B**) Assessment of cell viability in GC cells overexpressing Noxa via CCK8 assay. (**C**,**D**) Evaluation of the impact of Noxa overexpression on the clonogenic potential of GC cells using plate cloning assays. (**E**,**F**) Transwell assay depicting the influence of Noxa expression levels on the migratory and invasive capabilities of GC cells. (**G**,**H**) Scratch analysis illustrating the effect of Noxa expression levels on cell invasion ability. *P < 0.05; **P < 0.01; ***P < 0.001; ****P < 0.001.

To decipher the fuction of Noxa, we selected AGS and MKN-28 cell lines for Noxa overexpression and HGC-27 cell line for Noxa silencing. Subsequently, the transfection efficiency was verified via Western blot analysis, revealing a substantial increase in Noxa expression in AGS and MKN-28 cells (Fig. 2A) and a corresponding decrease in HGC-27 cells. Among the various knockdown approaches, minor interference 1 demonstrated the most pronounced efficacy (Fig. S2A). To probe the impact of Noxa on cellular proliferation, we conducted both CCK8 assays (Fig. 2B) and plate cloning experiments (Fig. 2C,D). These assays demonstrated that Noxa overexpression exerted a significant dampening effect on the proliferative capacity of AGS and MKN-28 cells in comparison to control groups. Conversely, Noxa knockdown in HGC-27 cells elicited the opposite response (Fig. S2B,C). These findings confirmed the inhibitory effect of Noxa on GC cell proliferation in vitro. We further delved into the influence of Noxa on cell migration and invasion through transwell and scratch assays. The results of the Transwell assay corroborated our expectations, revealing that Noxa overexpression substantially mitigated the metastatic potential of AGS and MKN-28 cells (Fig. 2E,F). Complementing this, the scratch assay gave additional evidence that Noxa overexpression inhibited the metastatic capabilities of gastric cancer cells (Fig. 2G,H). In stark contrast, the silencing of Noxa in HGC-27 cells promoted the migration and invasion of GC cells (Fig. S2D,E). Collectively, these results substantiate the role of Noxa in inhibiting the in vitro migration and invasion of GC cells. In summary, our results showed that Noxa prevented the progression of GC cells in vitro.

### Noxa is a target of hsa-miR-200b-3p in GC

**Figure 3:**
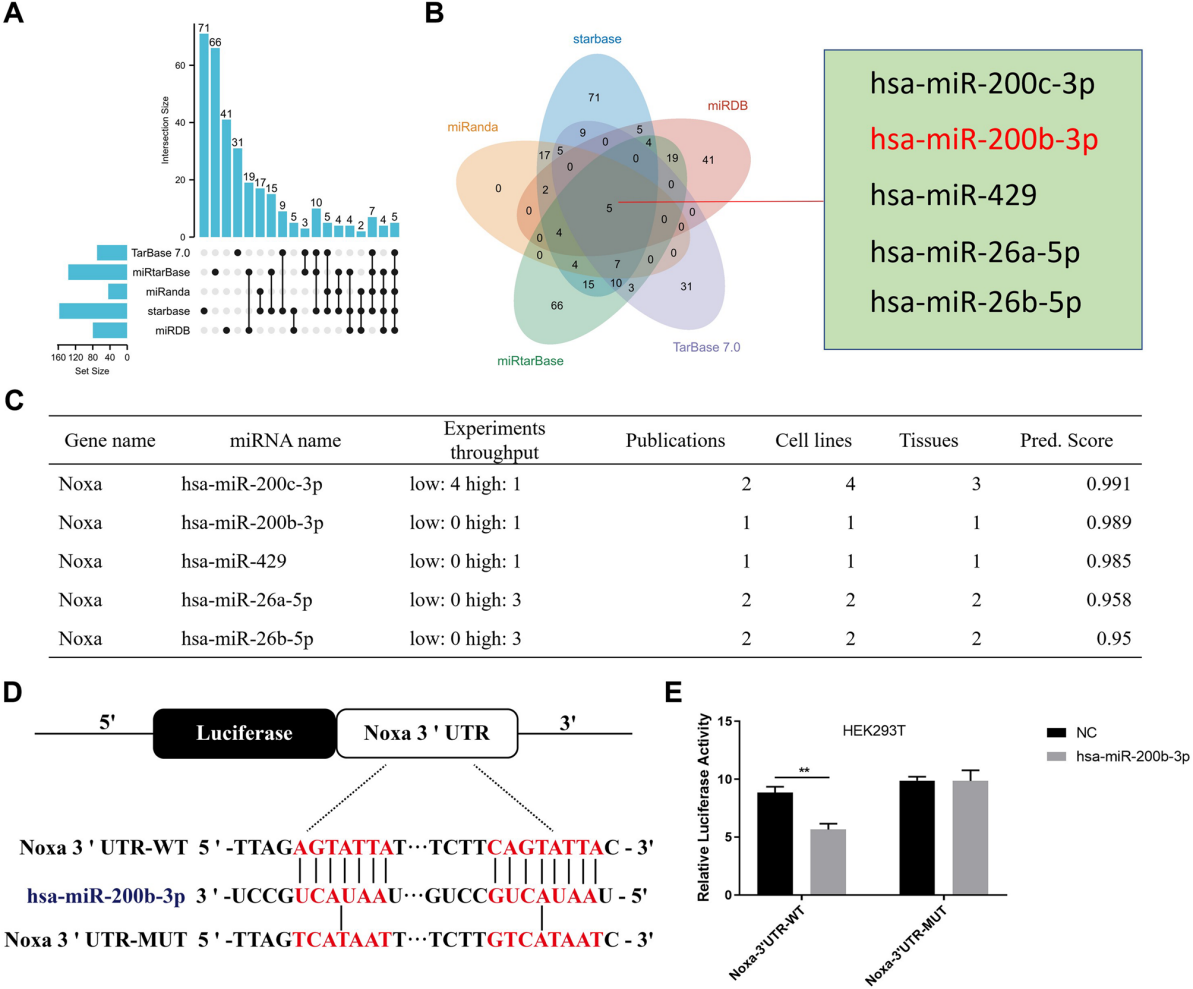
Noxa as a target of hsa-miR-200b-3p in GC. (**A**,**B**) Upset plots and Venn diagrams illustrate the potential target miRNAs predicted for Noxa. (**C**) TarBase 7.0 scores for the five co-predicted miRNAs are displayed. (**D**) Schematic representation of the binding site of hsa-miR-200b-3p within the Noxa 3' untranslated region. (**E**) Co-transfection of pcDNAmiR-200b-3p expression plasmid or negative control into HEK293T cells, followed by measurement of the relative activity of firefly luciferase.

After determining Noxa's crucial function in the suppression of cancer, we next investigated the upstream regulatory mechanism governing Noxa. As widely acknowledged, miRNAs exert their biological influence by orchestrating the regulation of target genes. In our pursuit, we harnessed the predictive potential of online platforms such as TarBase 7.0, miRanda, miRanda, starbase and miRDB to foretell potential miRNAs and took the intersection of the predicted results. The Upset and Venn diagrams showed the prediction results, in which there were five target miRNAs jointly predicted by the five databases, including hsa-miR-200c-3p, hsa-miR-200b-3p, hsa-miR-429, hsa-miR-26a-5p and hsa-miR-26b-5p (Fig. 3A,B). On this basis, the co-predicted five miRNAs were scored using the online website TarBase 7.0. Therefore, we opted to prioritize has-miR-200c-3p and hsa-miR-200b-3p as potential microRNAs candidate for the regulation of Noxa (Fig. 3C). miRNAs, which are small, tissue-specific RNAs, play a crucial role in maintaining cellular homeostasis by downregulating gene expression post-transcriptionally through binding to complementary sequences in their target mRNAs^31,32^. Minn's study^33^ indicates that the expression of miR-200b-3p is elevated in gastric cancer, while Bibi's research^34^ shows a decreased expression of miR-200c-3p in gastric cancer, which is consistent with our PCR validation results in 48 pairs of gastric cancer samples (Fig. S3A,B). The upregulation of miR-200b-3p, in particular, suggests its oncogenic role in gastric cancer, prompting us to focus on hsa-miR-200b-3p as a key regulator of Noxa. The potential binding sites of Noxa with hsa-miR-200b-3p were further predicted using TargetScan and miRDB online tools, which indicated that the 3′UTR region of Noxa had a potential complementary site in the seed region of hsa-miR-200b-3p (Fig. 3D). Further research into the direct binding of hsa-miR-200b-3p and Noxa was done using luciferase reporter assays. In this assay, we amplified and inserted both the wild-type (WT) and corresponding mutant (MT) versions of the Noxa 3′ UTR downstream of the luciferase reporter gene within the pmirGLO base vector. Remarkably, when pcDNAmiR-200b-3p was co-transfected with Noxa 3′ UTR-WT in HEK293T cells, a significant reduction in luciferase activity was observed compared to cells transfected with Noxa 3′ UTR-WT alone. Conversely, co-transfection of Noxa 3′ UTR-MT and pcDNAmiR-200b-3p showed no discernible difference in luciferase activity (Fig. 3E). Collectively, theseus findings substantiated that Noxa indeed functioned as a bona fide target of hsa-miR-200b-3p in gastric cancer cells.

### Suppression of Noxa by hsa-miR-200b-3p explains its anticancer activity in gastric cancer

**Figure 4:**
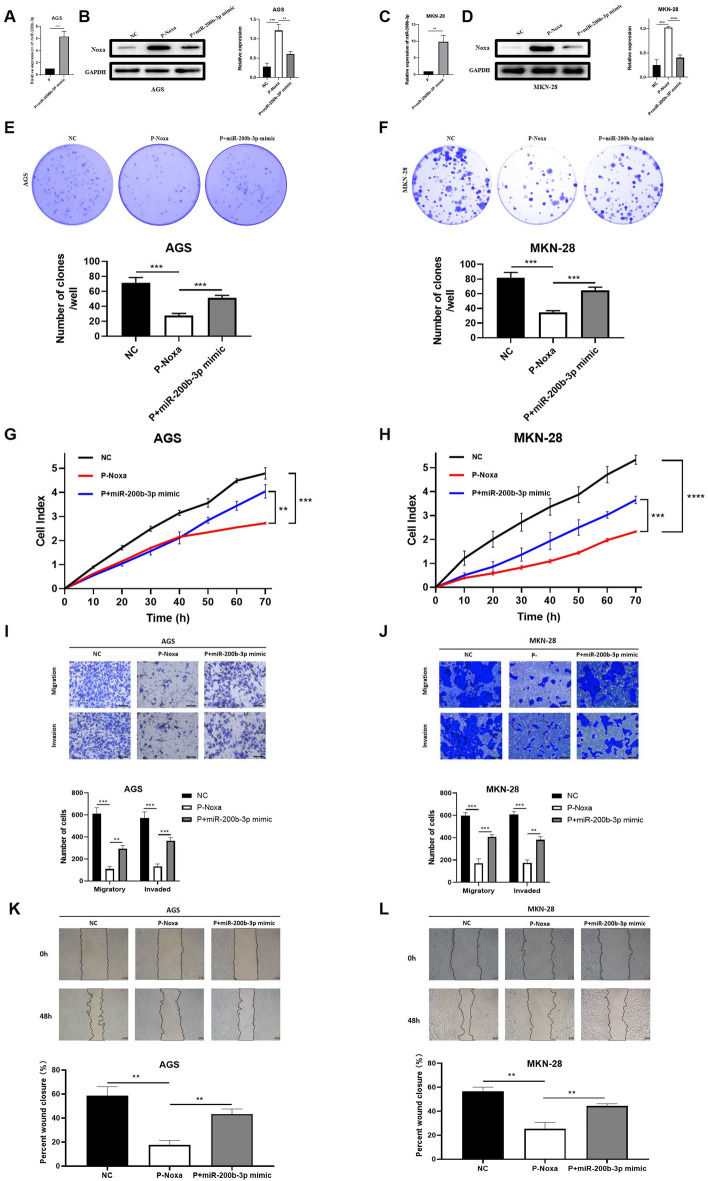
miR-200b-3p negatively regulates Noxa and thus gastric cancer progression. (**A**,**C**) Validation of miR-200b-3p levels after transfection of AGS and MKN-28 cells of P-Noxa group using miR-200b-3p mimic or negative control vector. (**B**,**D**) Validation of Noxa levels in GC cells of NC group, P-Noxa group and P + miR-200b-3p mimic groups. (**E**,**F**) Examination of plate cloning assay results for GC cells across the NC, P-Noxa, and P + miR-200b-3p mimic groups. (**G**,**H**) Analysis of RTCA assay data for GC cells within the NC, P-Noxa, and P + miR-200b-3p mimic groups. (**I**,**J**) Transwell assay evaluation of GC cell migration in the NC, P-Noxa, and P + miR-200b-3p mimic groups. (**K**,**L**) Scrutiny of GC cell migration using the scratch assay method within the NC, P-Noxa, and P + miR-200b-3p mimic groups.

To delve deeper into whether hsa-miR-200b-3p could promote gastric cancer (GC) growth by impeding Noxa, We initially tested whether miR-200b-3p might reverse Noxa's impact in GC cells. We initiated a targeted transfection procedure, introducing hsa-miR-200b-3p mimics into GC cells previously engineered to overexpress Noxa. The success of the transfection process was meticulously verified via qRT-PCR (Fig. 4A,C). These results served as a robust confirmation of the heightened levels of hsa-miR-200b-3p within the transfected cells. Furthermore, we conducted Western blot analysis to assess Noxa protein levels (Fig. 4B,D). These results revealed a significant reduction in Noxa protein expression upon overexpression of hsa-miR-200b-3p, providing substantial evidence to support the notion that Noxa is indeed a confirmed target of hsa-miR-200b-3p. Subsequently, our investigation ventured into the realm of cell proliferation dynamics. Plate cloning experiments confirmed that miR-200b-3p overexpression dramatically increased the rate of proliferation in AGS and MKN-28 cells, in sharp contrast to control cells (Fig. 4E,F). The congruence of these findings was further corroborated by our Real-Time Cell Analysis (RTCA) proliferation curves (Fig. 4G,H). The succeeding phase of our inquiry was devoted to elucidating the potential impact of this regulatory axis on the GC cells' capacity for invasion and migration. As anticipated, Noxa-overexpressing AGS and MKN-28 cells displayed markedly curtailed migratory and invasive abilities compared to their negative control counterparts. However, in an intriguing turn of events, upon ZNF519 down-regulation, the attenuated capacities of GC cells experienced a remarkable resurgence (Fig. 4I–L). In summary, these findings eloquently advocate for the potency of hsa-miR-200b-3p in negative regulation over Noxa, subsequently rekindling the proliferation, migration, and invasion attributes of GC cells.

### Noxa affects gastric cancer progression by regulating ZNF519

**Figure 5:**
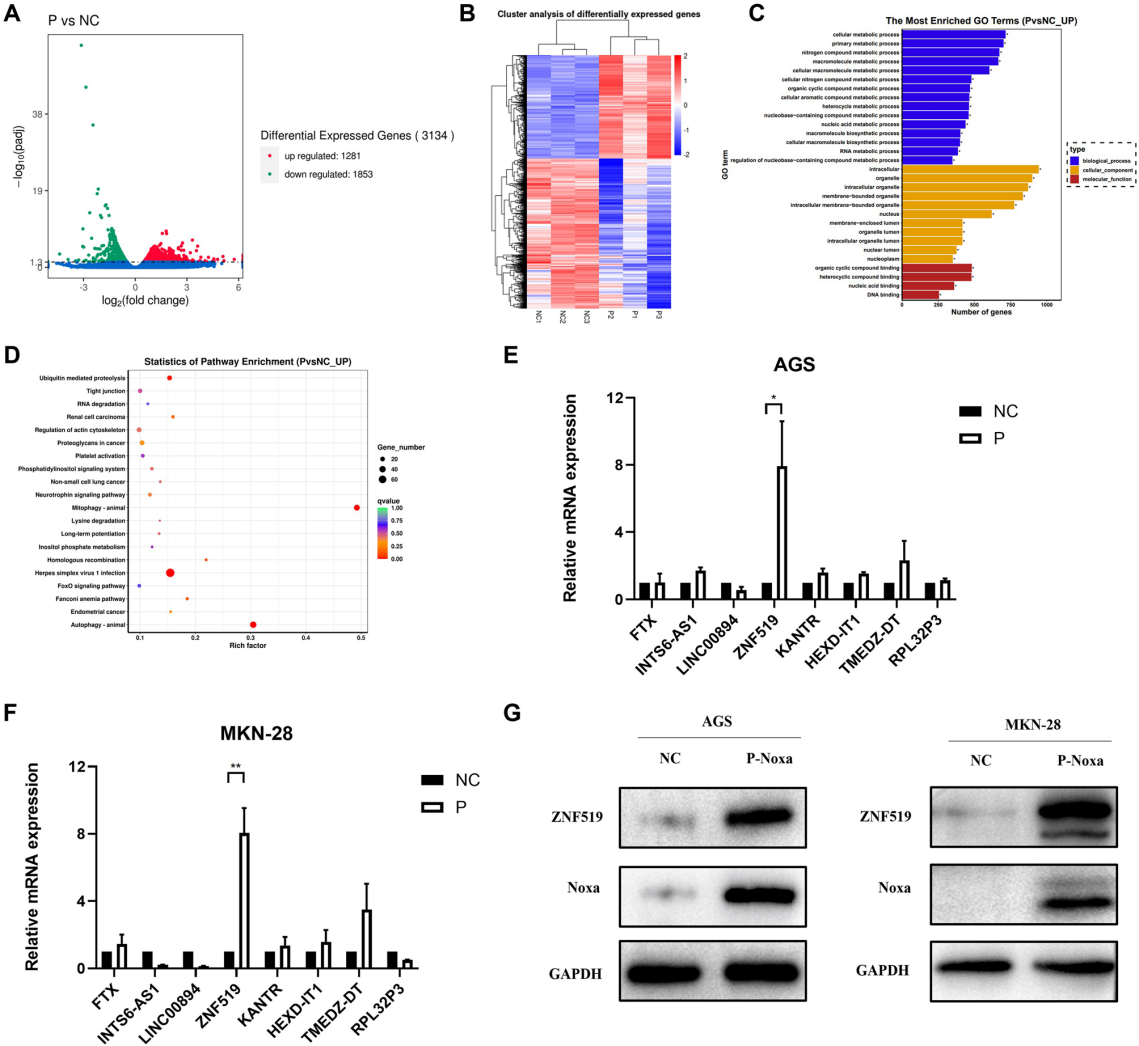
Analysis of Downstream Differential Proteins. (**A**) Volcano plot illustrating the distribution of differential proteins. (**B**) Heatmap depicting the differential protein expression patterns observed in transcriptome sequencing between AGS NC and AGS P-Noxa groups. (**C**,**D**) Enrichment analysis based on GO and KEGG pathways. (**E**,**F**) Verification of the transcript levels of the downstream differential gene, ZNF519. (**G**) Validation of the protein levels of the downstream differential gene, ZNF519.

We used transcriptome sequencing on two stably transfected cell lines, the AGS NC group and AGS P-Noxa group, with the aim of identifying the pertinent signaling pathways (*P*_adj_ < 0.05) in order to understand how Noxa affects the development of GC. Our sequencing analysis unveiled significant differences in gene expression profiles caused by the overexpression of the Noxa gene. Specifically, we identified 1281 genes that were up-regulated and 1853 genes that were down-regulated (Fig. 5A,B). Upon subjecting the data to GO and KEGG pathway^35^ analysis, we observed a notable enrichment in the Mitophagy-animal pathway resulting from Noxa overexpression (Fig. 5C,D). Within this enriched mitophagy pathway, we identified seven downstream proteins that consistently exhibited log2FC > 1, namely HEXD-IT1, LINC00894, INTS6-AS1, FTX, KANTR, ZNF519, and RPL32P3 (Table S3). According to our PCR findings, there is a sizable difference in ZNF519 expression between the NC and P-Noxa groups, prompting us to hypothesize that Noxa might exert its influence on the gastric cancer phenotype by regulating ZNF519 (Fig. 5E,F). This hypothesis was further substantiated by protein imprinting analysis, which confirmed a substantial increase in ZNF519 levels following Noxa overexpression (Fig. 5G). These results combined implied that Noxa played a pivotal role in modulating GC development by regulating ZNF519.

### Noxa inhibits the progression of GC cells by promoting ZNF519 expression

**Figure 6:**
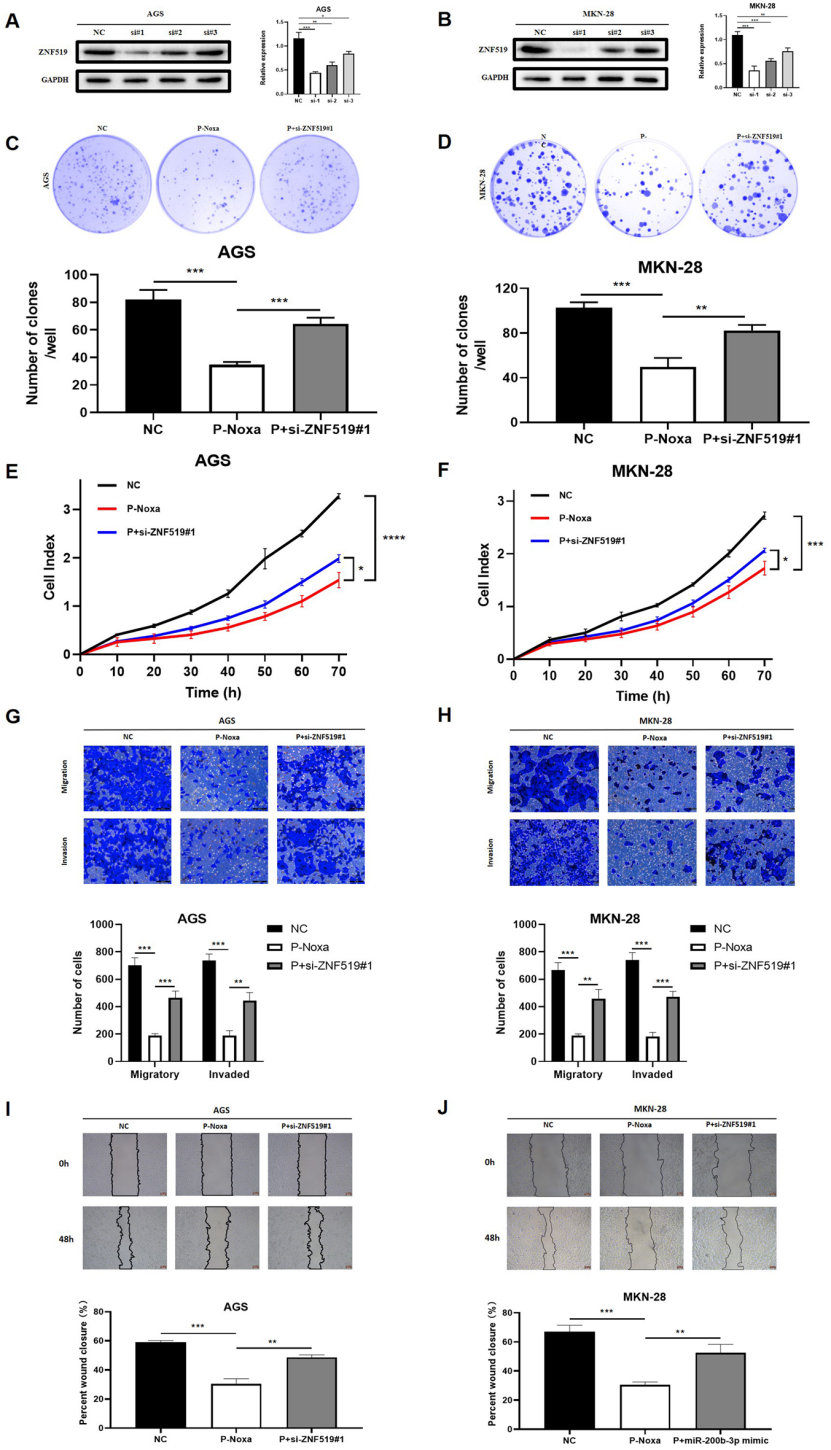
Rescue of gastric cancer proliferative capacity through ZNF519 silencing. (**A**,**B**) Validation of efficiency following the transfection of AGS and MKN-28 cells with ZNF519 siRNA. (**C**,**D**) Analysis of plate cloning assay results for GC cells in the NC, P-Noxa, and P + si-ZNF519#1 groups. (**E**,**F**) RTCA assay assessment of GC cells in the NC, P-Noxa, and P + si-ZNF519#1 groups. (**G**,**H**) Transwell assay analysis of GC cells in the NC group, P-Noxa group, and P + si-ZNF519#1 group. (**I**,**J**) Evaluation of scratch assay results for GC cells in the NC group, P-Noxa group, and P + si-ZNF519#1 group, illustrating the migratory potential of these cells.

To further investigate whether Noxa's impact on GC cell proliferation is mediated through its regulation of ZNF519 expression, we We carried out several rescue experiments. We employed three different siRNAs to silence ZNF519 in GC cells and confirmed the transfection efficiency via Western Blot. Among the siRNAs tested, siRNA#1 demonstrated the highest effectiveness (Fig. 6A,B). Plate cloning results indicated that the proliferative capacity of the Noxa-overexpressing group (P-Noxa) was notably decreased in comparison to the negative control group. Therefore, we introduced ZNF519 interference (P + si-ZNF519#1) based on our prior findings. As anticipated, the proliferation of gastric cancer cells was restored upon downregulating ZNF519 (Fig. 6C,D). Subsequently, we employed RTCA proliferation assays to investigate whether a similar approach would yield parallel results. It was evident that the proliferation rate of the P-Noxa group was notably reduced in comparison to the negative control group, but the proliferation of gastric cancer cells was reinstated following ZNF519 silencing (Fig. 6E,F). These results collectively suggest that Noxa modulates the proliferative capacity of GC cells by regulating ZNF519 expression. We then delved into whether this treatment could influence the migratory and invasive abilities of GC cells, employing Transwell and scratch assays. As expected, AGS and MKN-28 cells overexpressing Noxa exhibited markedly reduced migratory and invasive capabilities compared to the negative control group. However, these abilities were reinstated upon downregulation of ZNF519 in GC cells (Fig. 6G–J). In summary, our findings indicate that Noxa exerts control over the progression of GC cells through its regulatory influence on ZNF519 expression.

### The abundance of ZNF519 was regulated by the miR-200b-3p/Noxa axis in GC cells

**Figure 7:**
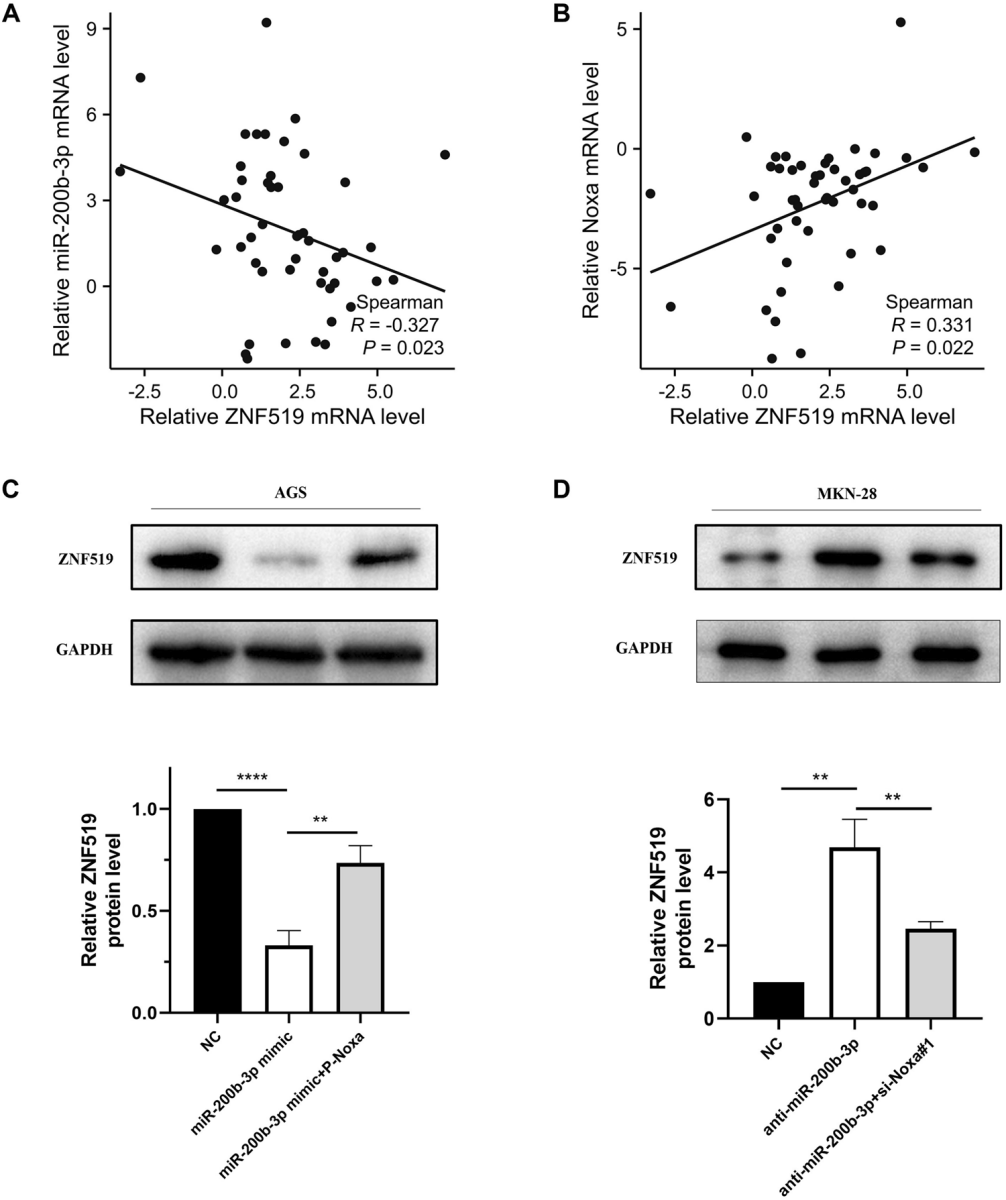
The abundance of ZNF519 is modulated by the miR-200b-3p/Noxa axis. (**A**,**B**) The linear association between the mRNA levels of ZNF519 and miR-200b-3p or Noxa was evaluated by Spearman's correlation coefficient in GC cells. (**C**) Western blot was performed to determine the protein level of ZNF519 in AGS cells transfected with vector NC, miR-200b-3p mimic, or miR-200b-3p mimic + P-Noxa. (**D**) Western blot analysis was performed in MKN-28 cells transfected with vector NC, anti-miR-200b-3p, or anti-miR-200b-3p + si-Noxa#1 to evaluate the protein level of ZNF519.

We harnessed the Spearman correlation coefficient (n = 48) to delve into the relationships among ZNF519 mRNA, miR-200b-3p, and Noxa in GC tissue. Our investigations unveiled a significant although weak positive correlation between ZNF519 mRNA and miR-200b-3p (R = − 0.327), while conversely, a significant although weak negative correlation emerged between ZNF519 mRNA and Noxa (R = 0.331) (Fig. 7A,B). To shed light on the intricate connections linking ZNF519, miR-200b-3p, and Noxa, we embarked on transfection assays using AGS and MKN-28 cells, grouped into three categories. Overexpression of miR-200b-3p led to a reduction in ZNF519 protein expression, while co-transfection of miR-200b-3p and P-Noxa brought about an increase in ZNF519 protein levels (Fig. 7C). Furthermore, AGS and MKN-28 cells underwent simultaneous transfection with anti-miR-200b-3p and si-Noxa. Deleting miR-200b-3p resulted in the restoration of ZNF519 protein levels. In the case of ZNF519, si-Noxa#1 transfection led to the downregulation of ZNF519 (Fig. 7D). According to these comprehensive results, we posited that miR-200b-3p exerts upstream regulation over Noxa, ultimately culminating in the downregulation of ZNF519.

### Overexpression of Noxa attenuates gastric cancer tumor growth in vivo

**Figure 8:**
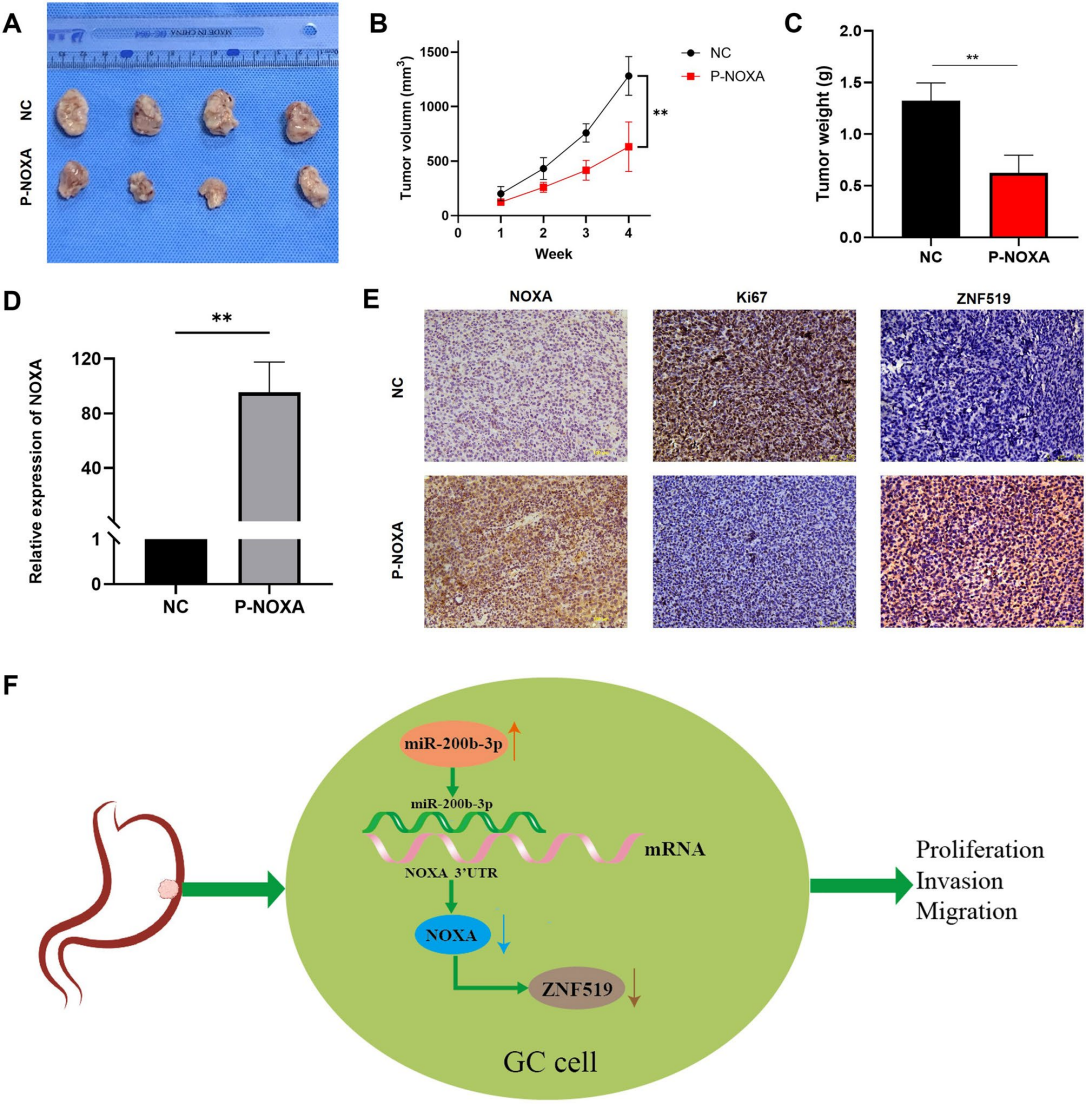
Overexpression of Noxa suppresses tumour growth of GC cells in vivo. (**A**) Impact of Noxa overexpression in AGS on gastric cancer tumorigenesis in vivo. (**B**) Quantification of subcutaneous tumor volume in each group (n = 4). (**C**) Assessment of nude mice weight before and after tumor formation in each group. (D) Validation of relative Noxa expression via RNA extraction from tumor tissues. (**E**) Evaluation of protein expression levels of Noxa, Ki67, and ZNF519 in tumor tissues using tissue microarray. (**F**) Schematic diagram illustrating the hsa-miR-200b-3p-Noxa-ZNF519 axis in GC. Increased expression of hsa-miR-200b-3p leads to a reduction in Noxa expression, subsequently resulting in the downregulation of ZNF519. Ultimately, this axis regulates GC cell proliferation, invasion, migration, and tumorigenesis.

Our research intended to clarify Noxa's effect on tumor growth in vivo. To achieve this, we established stable transfections of AGS gastric cancer cells with GV208-Noxa for robust Noxa overexpression. Subsequently, female nude mice, aged 4 weeks, were subjected to subcutaneous inoculations of these stably transfected cells into their axillae. The dimensions of the tumors were diligently assessed on a weekly basis, and at the conclusion of a 28-day period, the mice were compassionately euthanized, and tumor specimens were meticulously collected for further analysis. Our findings unequivocally demonstrate that the tumors in the AGS-GV208-Noxa group were much smaller when compared with the NC group (Fig. 8A,B). It's worth noting that the AGS-GV208-Noxa group also exhibited a noticeable decline in body weight when contrasted with the control group (Fig. 8C). Upon RNA extraction from the lysed tumor tissues, we conducted qRT-PCR to ascertain the transfection efficiency. The results revealed a substantial upswing in Noxa expression within the AGS-GV208-Noxa group (Fig. 8D). Moreover, in the AGS-GV208-Noxa group, the intensity of Ki-67 staining and its expression levels were diminished, while the expression of Noxa and ZNF519 were amplified (Fig. 8E). We have presented a schematic representation illustrating the impact of the miR-200b-3p-Noxa-ZNF519 axis on gastric cancer (Fig. 8F). Collectively, these findings strongly indicate that Noxa exerts a potent inhibitory effect on tumor development in nude mice.

## Discussion

Gastric cancer represents one of the most lethal malignancies worldwide, posing a significant threat to the daily lives of people^36^. The inherent challenges of delayed diagnosis and the high propensity for metastasis in gastric cancer contribute to elevated mortality rates, with a median survival rate of less than 1 year for patients with metastatic gastric cancer^37^. Cancer cells exhibit marked genetic and epigenetic alterations, frequently instigating the initiation and progression of malignancies by perturbing the molecular and cellular processes governing cancer cells^38^. Consequently, a comprehensive exploration of the molecular and cellular mechanisms underpinning the pathogenesis of gastric cancer is imperative for precise diagnosis, prognostic assessment, and therapeutic strategies. Dysregulation of oncogenes constitutes a pivotal element in tumorigenesis, including gastric cancer (GC). In our pursuit of enhancing the survival outcomes for gastric cancer patients, it is imperative to uncover novel molecules implicated in the GC phenotype. Within the scope of this investigation, we have unearthed that the protein known as fopperol-12-myristate-13-acetate-inducible protein-1, Noxa, exhibits diminished expression levels in GC tissues. Nevertheless, the precise role of Noxa in GC has hitherto remained enigmatic.

Our study highlights the function of Noxa and its mechanism in regulating GC proliferation and metastasis. Noxa expression has been shown to be dysregulated in tumours such as colorectal cancer^39^, pancreatic cancer^40^, human adenoid cystic carcinoma^41^, and breast cancer^42^.Noxa is now being tested as a promising therapeutic target in cancer biology. In our study, we observed a significant downregulation of Noxa in gastric cancer cell lines and tissues. Furthermore, we found that its overexpression had an inhibitory effect on gastric cancer cell proliferation, invasion, and migration. In GC tissues, low Noxa expression was correlated with advanced T stage and distant metastasis. Meanwhile, gastric cancer patients with low expression of Noxa had poorer prognosis, and it might serve as an independent predictor of GC patients' poor prognosis. Therefore, Noxa may be an effective predictor of GC carcinogenesis and prognosis.

miRNAs are small molecule non-coding RNAs found in eukaryotic organisms. miRNAs can bind to mRNA target genes and regulate post-transcriptional levels of gene expression^43^. miRNAs potentially functioned in cancer by manipulating the expression of tumour suppressor genes and oncogenes, and were involved in the regulation of cell proliferation, apoptosis, invasion/migration and angiogenesis^44^. miRNAs are widely known for their regulatory role in translation by targeting the 3′UTR region of mRNAs. In this study, we identified Noxa as the target of hsa-miR-200b-3p based on luciferase reporter assay. Enhancement of hsa-miR-200b-3p expression could be useful in restoring the inhibitory effects caused by Noxa in the progression of GC cells. Researchers reported that miR-200b-3p attenuated TNF-α-induced apoptosis in rat intestinal epithelial cells by negatively regulating KHDRBS1^45^. Liu et al. demonstrated that LncRNA XIST acted as a molecular sponge for miR-200b-3p to regulate ZEB1/2 and promote hepatocellular carcinoma proliferation, migration, and invasion^46^. Huang et al. demonstrated for the first time that LncRNA-SNHG29 prevents the calcification of vascular smooth muscle cells by upregulating the α-Klotho/FGFR1/FGF23 axis via downregulating miR-200b-3p^47^. Considering that hsa-miR-200b-3p can bind to the 3′-UTR of Noxa mRNA and affects Noxa transcription after binding. Meanwhile, the increased expression of hsa-miR-200b-3p was found to result in the downregulation of Noxa, further suggesting that miR-200b-3p could manage Noxa. In this study, we reported that miR-200b-3p could suppress Noxa and exerting regulatory control over the progression of gastric cancer through the direct inhibition of Noxa expression.

Tumour regulatory mechanisms are complex and diverse. In this study, RNA-seq technology was used to analyze the expression and regulation of the transcriptome. Using RNA-seq methods, mRNA was extracted from total RNA, reverse transcribed into cDNA, and sequenced to identify and quantify transcripts. Downstream RNA-seq analysis is an effective method to identify downstream targets associated with signalling pathways and helps to gain insight into the complexity and diversity of gene regulatory networks^48^. RNA-seq results showed that overexpression of the Noxa gene altered the ZNF519 and Mitophagy-animal signalling pathways thereby affecting the progression of GC cells. ZNF519's regulation of typical physiological functions is not entirely understood, but it has been demonstrated the involvement of ZNF519 in the development of various types of tumors. Fan et al. showed that one of the hub genes linked to long-term survival in breast cancer is ZNF519^49^. Sui et al. showed that ZNF519 may be actively involved in adaptive colonisation of bone metastatic breast cancer cells^50^. In our study, we detected Noxa-induced disruption of the Mitophagy-animal signalling pathway, and further confirmed by in vitro experiments that Noxa could inhibit GC proliferation and invasion via ZNF519. These results indicate that ZNF519 is commonly expressed in tumour cells and the degree of tumor aggressiveness is directly correlated with ZNF519 expression.

In conclusion, Our work supports Noxa to be the possible new diagnostic biomarker and therapeutic target for GC. The newly discovered Noxa inhibits the progression of gastric cancer cells by regulating the expression of ZNF519, which provides a theoretical basis for revealing the pathogenesis of GC and developing potential target therapies for GC.

## Conclusions

In gastric cancer, Noxa expression is markedly diminished compared to that observed in normal gastric tissues and cells. Patients with low Noxa expression tend to present with advanced T3 or T4 stage disease, as well as distant metastasis. Noxa has demonstrated its ability to impede the progression of gastric cancer by effectively curtailing the metastatic potential and proliferation of gastric cancer cells, both in vitro and in vivo. It is worth noting that miR-200b-3p plays a pivotal role in downregulating the expression of Noxa, thereby fostering the metastasis and proliferation of gastric cancer cells. Noxa operates to stimulate ZNF519 in the downstream autophagy-animal signaling pathway, ultimately suppressing the advancement of gastric cancer cells. The hsa-miR-200b-3p/Noxa/ZNF519 axis furnishes a solid theoretical foundation for unraveling the pathogenesis of gastric cancer and presents a promising avenue for the development of potential therapeutic targets in the fight against this disease.

### Supplementary Information

Supplementary Figure S1.

Supplementary Figure S2.

Supplementary Figure S3.

Supplementary Table S1.

Supplementary Table S2.

Supplementary Table S3.

Supplementary Information.

Supplementary Legends.

## Data Availability

The datasets used and/or analysed during the current study are available from the corresponding author on reasonable request.
