# Supplementary figures and images for: Noxa inhibits oncogenesis through ZNF519 in gastric cancer and is suppressed by hsa-miR-200b-3p
Source: Sci Rep. 2024 Mar 19;14:6568. doi: 10.1038/s41598-024-57099-7 (PMC10951337; doi:10.1038/s41598-024-57099-7)

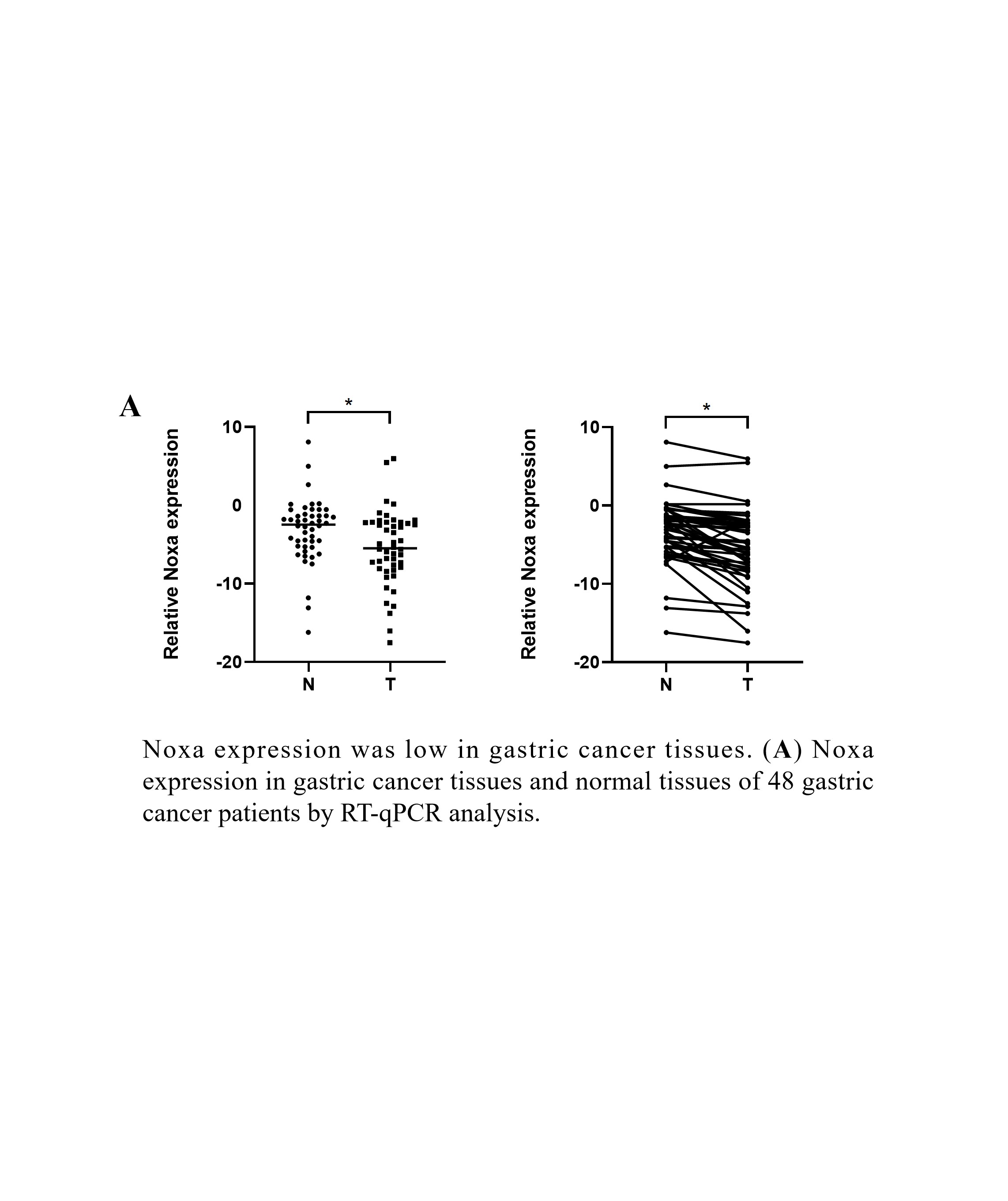

Supplement: Supplementary file 1 — Supplementary Figure S1. [file 41598_2024_57099_MOESM1_ESM.jpg]

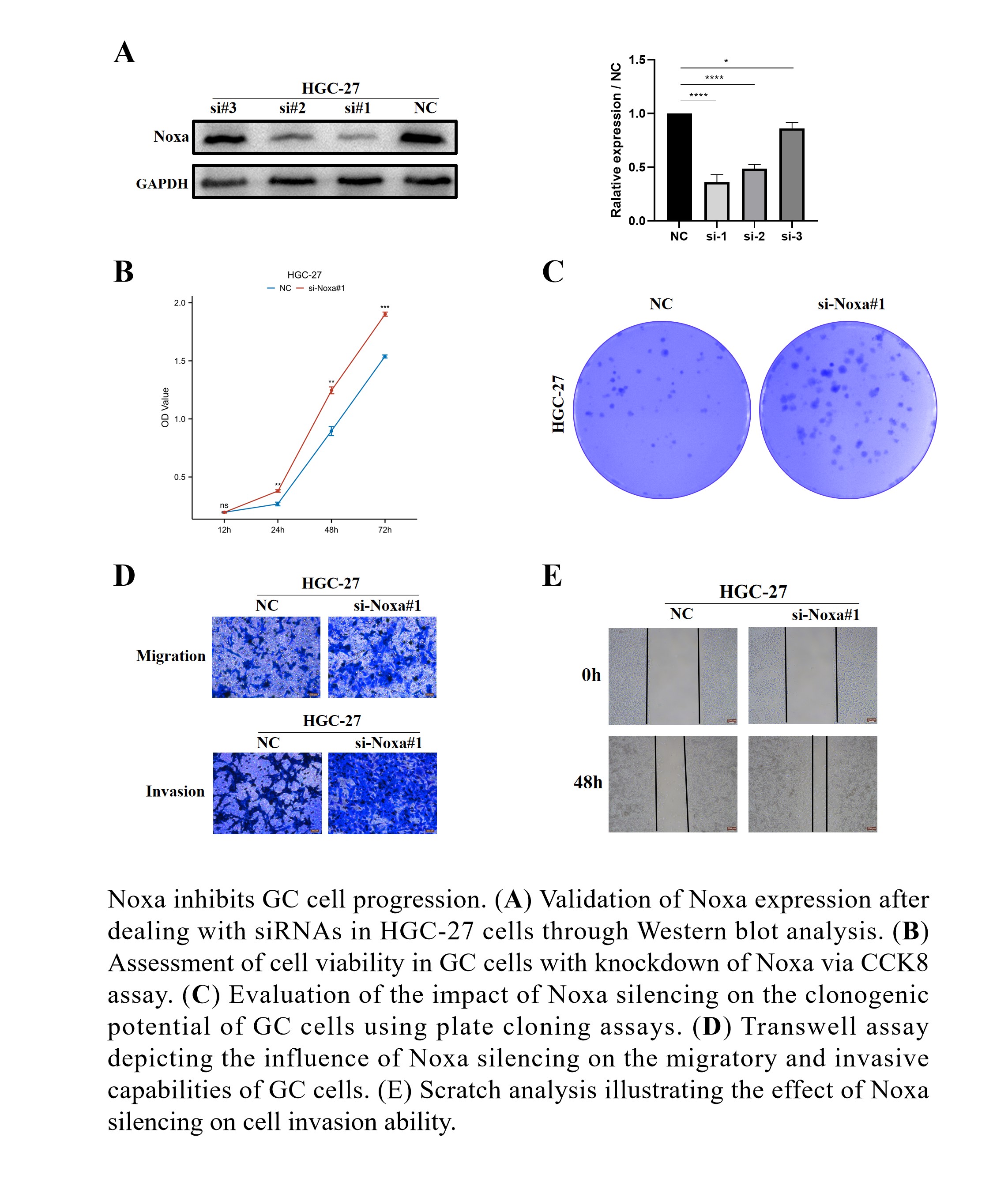

Supplement: Supplementary file 2 — Supplementary Figure S2. [file 41598_2024_57099_MOESM2_ESM.jpg]

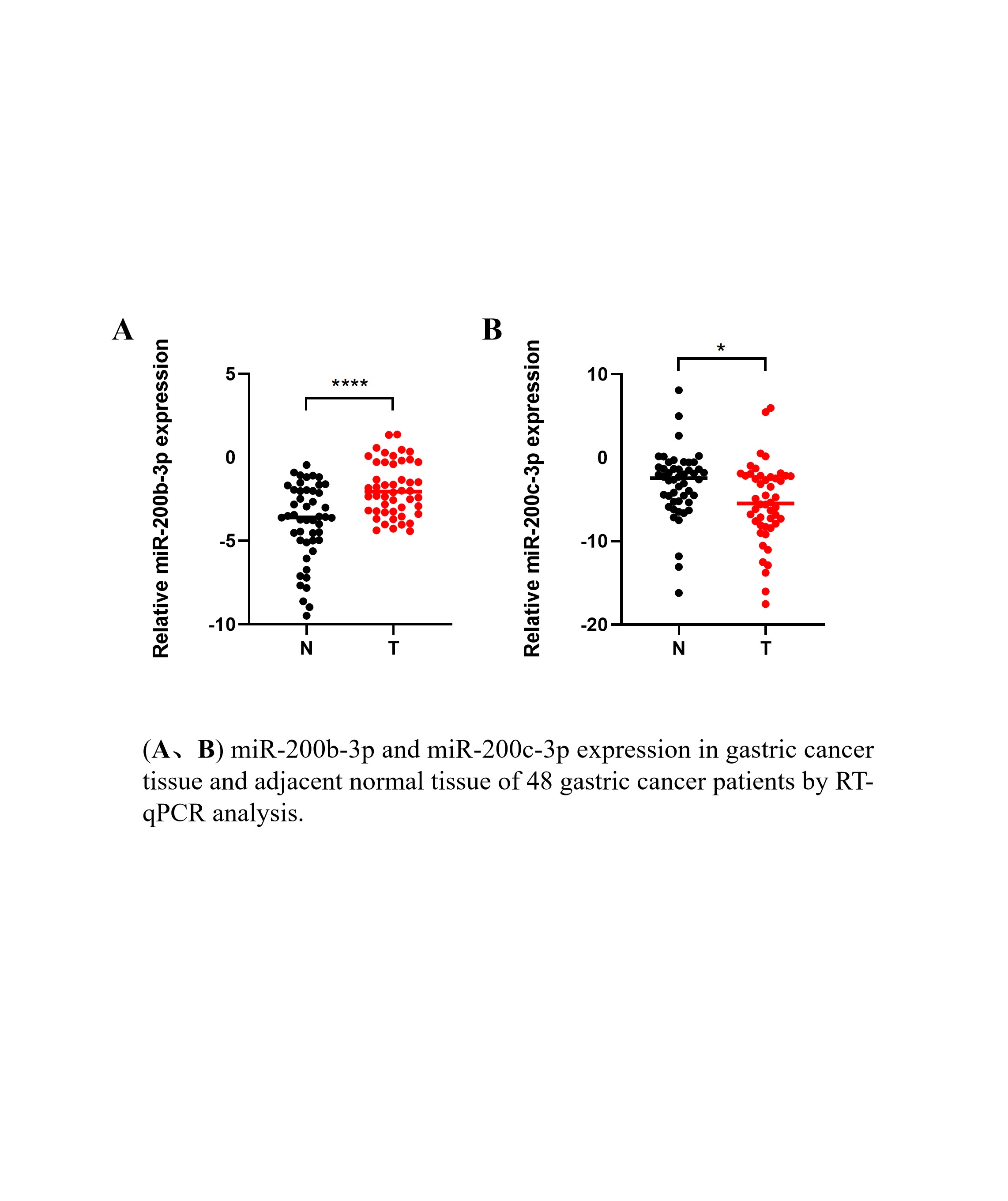

Supplement: Supplementary file 3 — Supplementary Figure S3. [file 41598_2024_57099_MOESM3_ESM.jpg]
