# Supplementary material for: Noxa inhibits oncogenesis through ZNF519 in gastric cancer and is suppressed by hsa-miR-200b-3p
Source: Sci Rep. 2024 Mar 19;14:6568. doi: 10.1038/s41598-024-57099-7 (PMC10951337; doi:10.1038/s41598-024-57099-7)
Supplement: Supplementary file 4 — Supplementary Table S1. [file 41598_2024_57099_MOESM4_ESM.docx]

| Name | Sequence ( 5' → 3' ) |
| --- | --- |
| hZNF519 si-1 sense | GCAAGGCAUACAAGAUUCAUUTT |
| hZNF519 si-1 antisense | AAUGAAUCUUGUAUGCCUUGCTT |
| hZNF519 si-2 sense | GGAGACAAAGAAUAUAGAATT |
| hZNF519 si-2 antisense | UUCUAUAUUCUUUGUCUCCTT |
| hZNF519 si-3 sense | GACCCAUUCUCAAAGCUUATT |
| hZNF519 si-3 antisense | UAAGCUUUGAGAAUGGGUCTT |

| Name | Sequence ( 5' → 3' ) |
| --- | --- |
| hNoxa si-1 sense | UGGAAGUCGAGUGUGCUACUCTT |
| hNoxa si-1 antisense | GAGUAGCACACUCGACUUCCATT |
| hNoxa si-2 sense | CCGGCAGAAACUUCUGAAUTT |
| hNoxa si-2 antisense | AUUCAGAAGUUUCUGCCGGTT |
| hNoxa si-3 sense | CCAACUCAGCACAUUGUAUTT |
| hNoxa si-3 antisense | AUACAAUGUGCUGAGUUGGTT |

hsa-miR-200b-3p mimic sense UAAUACUGCCUGGUAAUGAUGA

hsa-miR-200b-3p inhibitor sense UCAUCAUUACCAGGCAGUAUUA
