# Supplementary material for: Noxa inhibits oncogenesis through ZNF519 in gastric cancer and is suppressed by hsa-miR-200b-3p
Source: Sci Rep. 2024 Mar 19;14:6568. doi: 10.1038/s41598-024-57099-7 (PMC10951337; doi:10.1038/s41598-024-57099-7)
Supplement: Supplementary file 5 — Supplementary Table S2. [file 41598_2024_57099_MOESM5_ESM.docx]

| Name | Sequence ( 5' → 3' ) |
| --- | --- |
| Noxa Forward primer | CCTGCAGGACTGTTCGTGTT |
| Noxa Reverse primer | CACTCGACTTCCAGCTCTGCT |
| GAPDH Forward primer | TCGGAGTCAACGGATTTGGT |
| GAPDH Reverse primer | TTCCCGTTCTCAGCCTTGAC |

| Name | Sequence ( 5' → 3' ) |
| --- | --- |
| hsa-miR-200b-3p Forward primer | TAATACTGCCTGGTAATGATGA |
| hsa-miR-200c-3p Forward primer | TAATACTGCCGGGTAATGATGGA |

| Name | Sequence ( 5' → 3' ) |
| --- | --- |
| HEXD-IT1 Forward primer | TAAAGGGAAGGCGAGGGTTA |
| HEXD-IT1 Reverse primer | AGGACCCAACTGCCTCATC |
| LINC00894 Forward primer | GGCAGTTCCTTCCCAATG |
| LINC00894 Reverse primer | CCTGTGGCTGCTGTTGTAGT |
| INTS6-AS1 Forward primer | TGTCTTTCCTTCAGCCTCGT |
| INTS6-AS1 Reverse primer | GTGGGAGAGGCCCTGTAAAT |
| FTX Forward primer | TTGGGAGAGGCTCAAATCAG |
| FTX Reverse primer | GACATTCATGGCAACCGTAA |
| KANTR Forward primer | AGCCAGTGTCCCTTACACAA |
| KANTR Reverse primer | GTTCCTATTCGGCCATCTTG |
| ZNF519 Forward primer | TTTCATTTGGCATCTGTGGA |
| ZNF519 Reverse primer | CGGAGCTGACAATGTGCTTA |
| RPL32P3 Forward primer | GGAGAACACGGATGAATGGT |
| RPL32P3 Reverse primer | CTTTGCAGGGTTGTCCTCTC |
