# Supplementary material for: Noxa inhibits oncogenesis through ZNF519 in gastric cancer and is suppressed by hsa-miR-200b-3p
Source: Sci Rep. 2024 Mar 19;14:6568. doi: 10.1038/s41598-024-57099-7 (PMC10951337; doi:10.1038/s41598-024-57099-7)
Supplement: Supplementary file 6 — Supplementary Table S3. [file 41598_2024_57099_MOESM6_ESM.docx]

| Gene_id | Gene name | readcount_P | readcount_NC | log2FoldChange | pval | padj |
| --- | --- | --- | --- | --- | --- | --- |
| ENSG00000279066 | HEXD-IT1 | 7.2540 | 0.0000 | Inf | 0.0023 | 0.0284 |
| ENSG00000235703 | LINC00894 | 39.1604 | 9.0386 | 2.1152 | 0.0008 | 0.0128 |
| ENSG00000236778 | INTS6-AS1 | 24.7746 | 7.0677 | 1.8096 | 0.0037 | 0.0397 |
| ENSG00000230590 | FTX | 316.7751 | 125.2860 | 1.3382 | 0.0000 | 0.0005 |
| ENSG00000232593 | KANTR | 105.7028 | 45.2537 | 1.2239 | 0.0001 | 0.0030 |
| ENSG00000175322 | ZNF519 | 109.0398 | 50.0273 | 1.1241 | 0.0002 | 0.0051 |
| ENSG00000251474 | RPL32P3 | 287.4590 | 134.2417 | 1.0985 | 0.0002 | 0.0047 |

Table 2 Genes with higher difference between NC and P-Noxa groups
