# Supplementary material for: Noxa inhibits oncogenesis through ZNF519 in gastric cancer and is suppressed by hsa-miR-200b-3p
Source: Sci Rep. 2024 Mar 19;14:6568. doi: 10.1038/s41598-024-57099-7 (PMC10951337; doi:10.1038/s41598-024-57099-7)
Supplement: Supplementary file 7 — Supplementary Information. [file 41598_2024_57099_MOESM7_ESM.pdf]

Figure 1(D) Immunoblotting experiments examining Noxa expression in 7 paired tissue samples

GAPDH

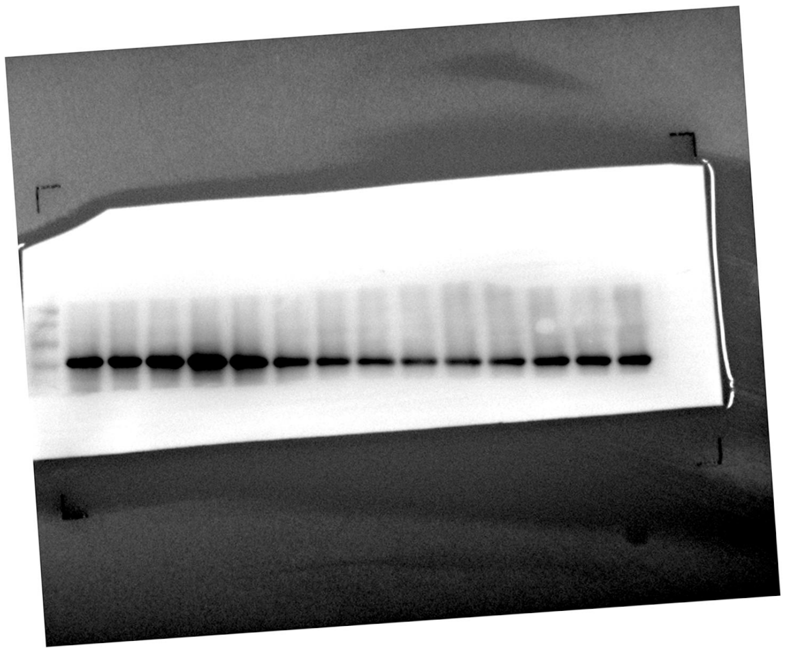

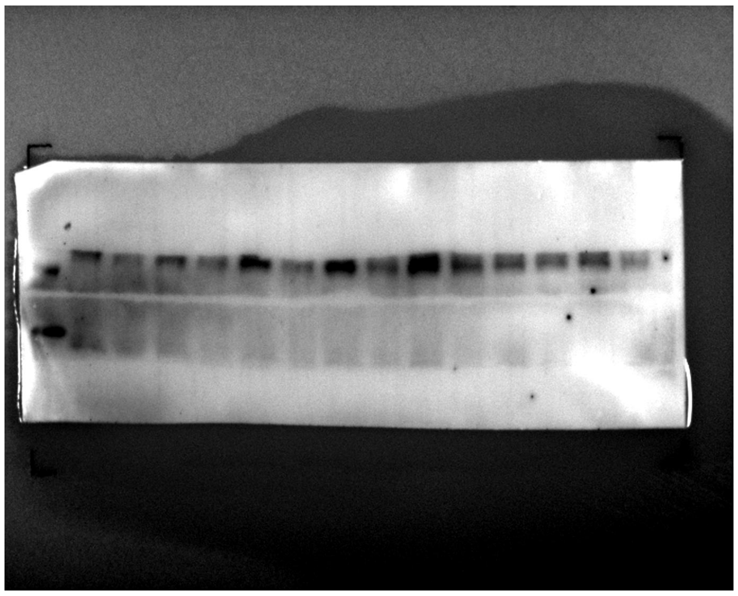

Noxa

GAPDH

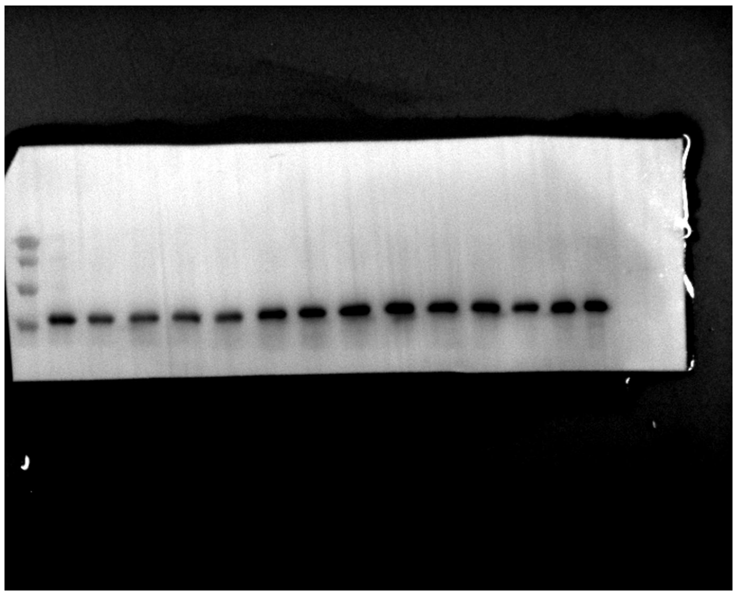

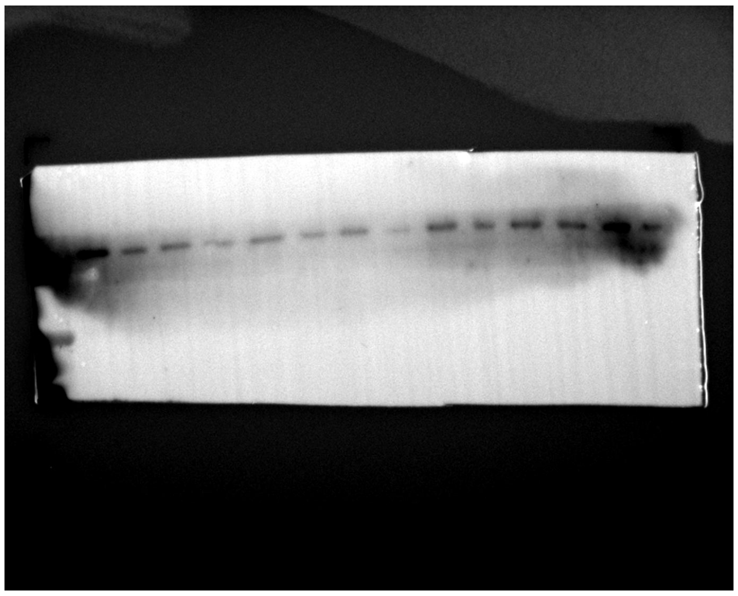

Noxa

GAPDH

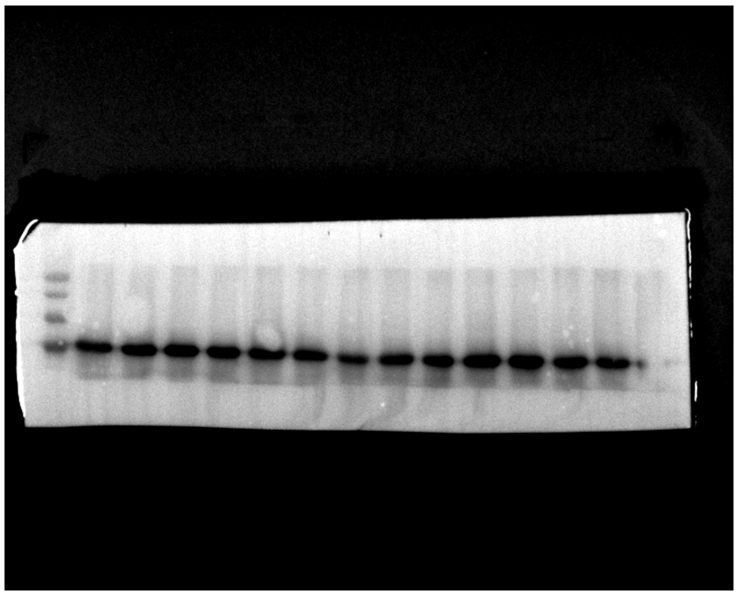

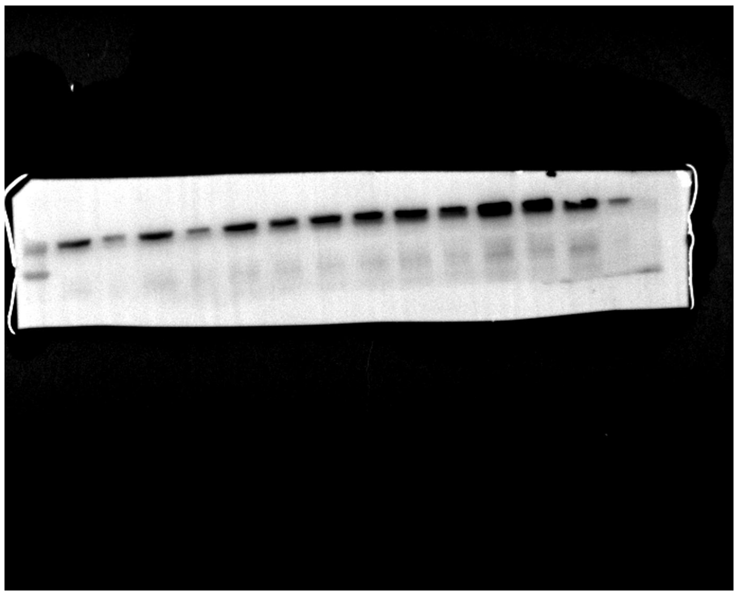

Noxa

Figure 1(F)Western blot analysis depicting Noxa gene expression levels in the normal gastric epithelial cell line GES-1 and gastric cancer cell lines AGS, HGC-27, and MKN-28

GAPDH

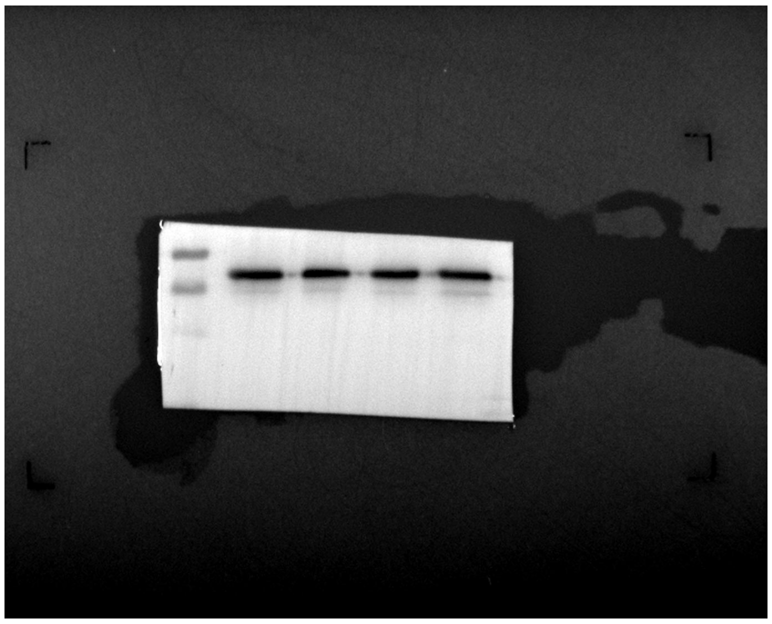

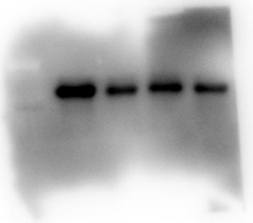

Noxa

GAPDH

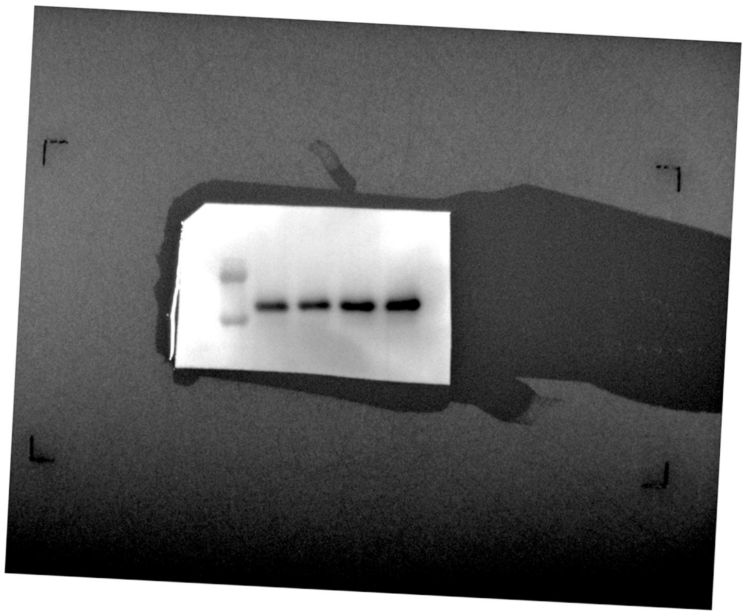

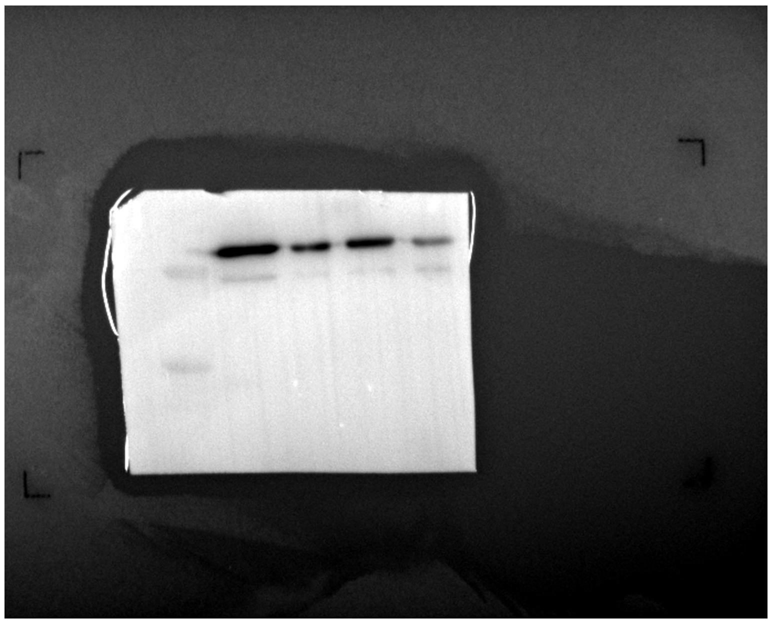

Noxa

GAPDH

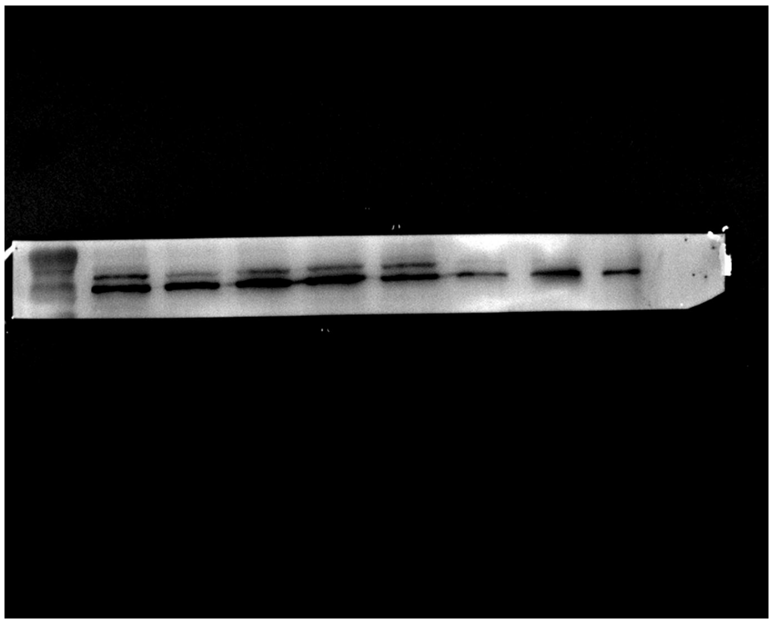

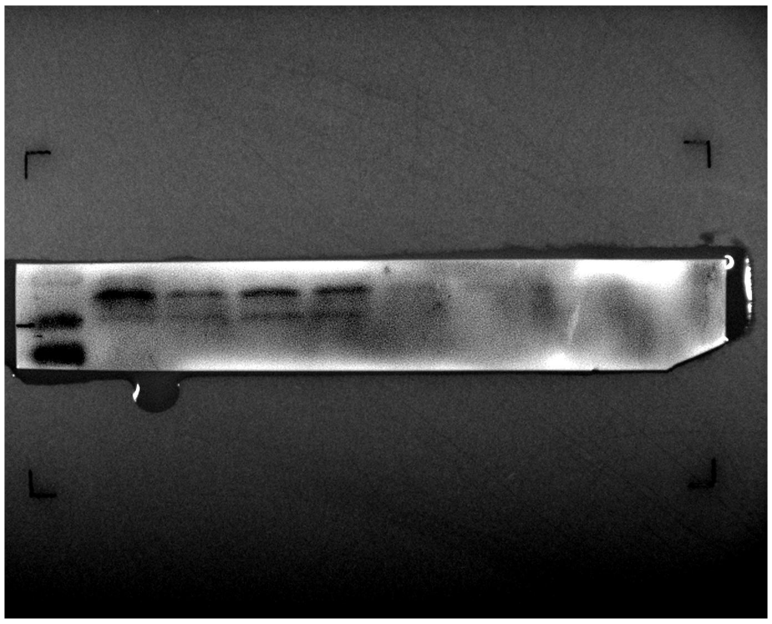

Noxa

Figure 2(A) Validation of Noxa overexpression in AGS cells through Western blot analysis

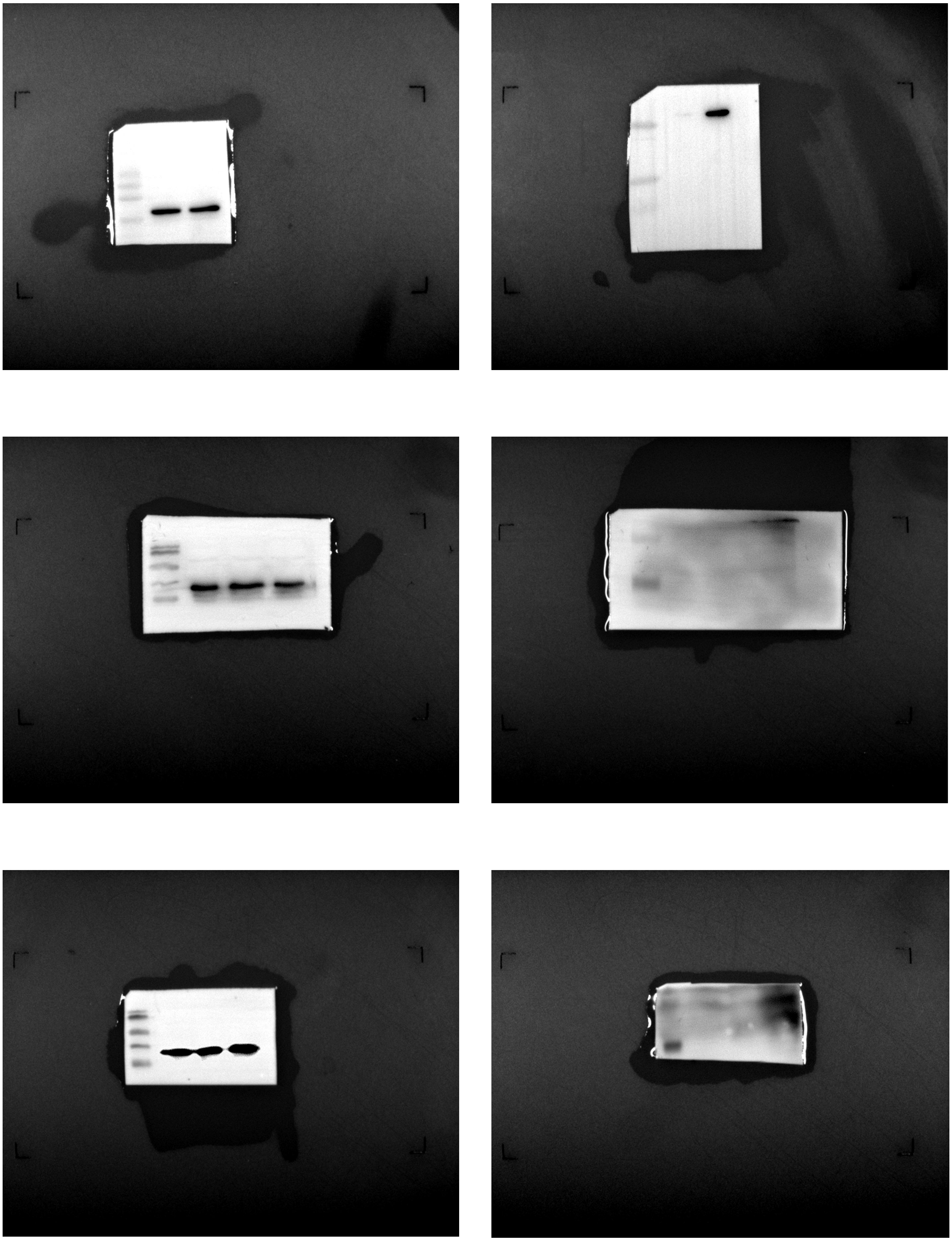

GAPDH Noxa

GAPDH Noxa

GAPDH Noxa

Figure 2(A) Validation of Noxa overexpression in MKN-28 cells through Western blot analysis

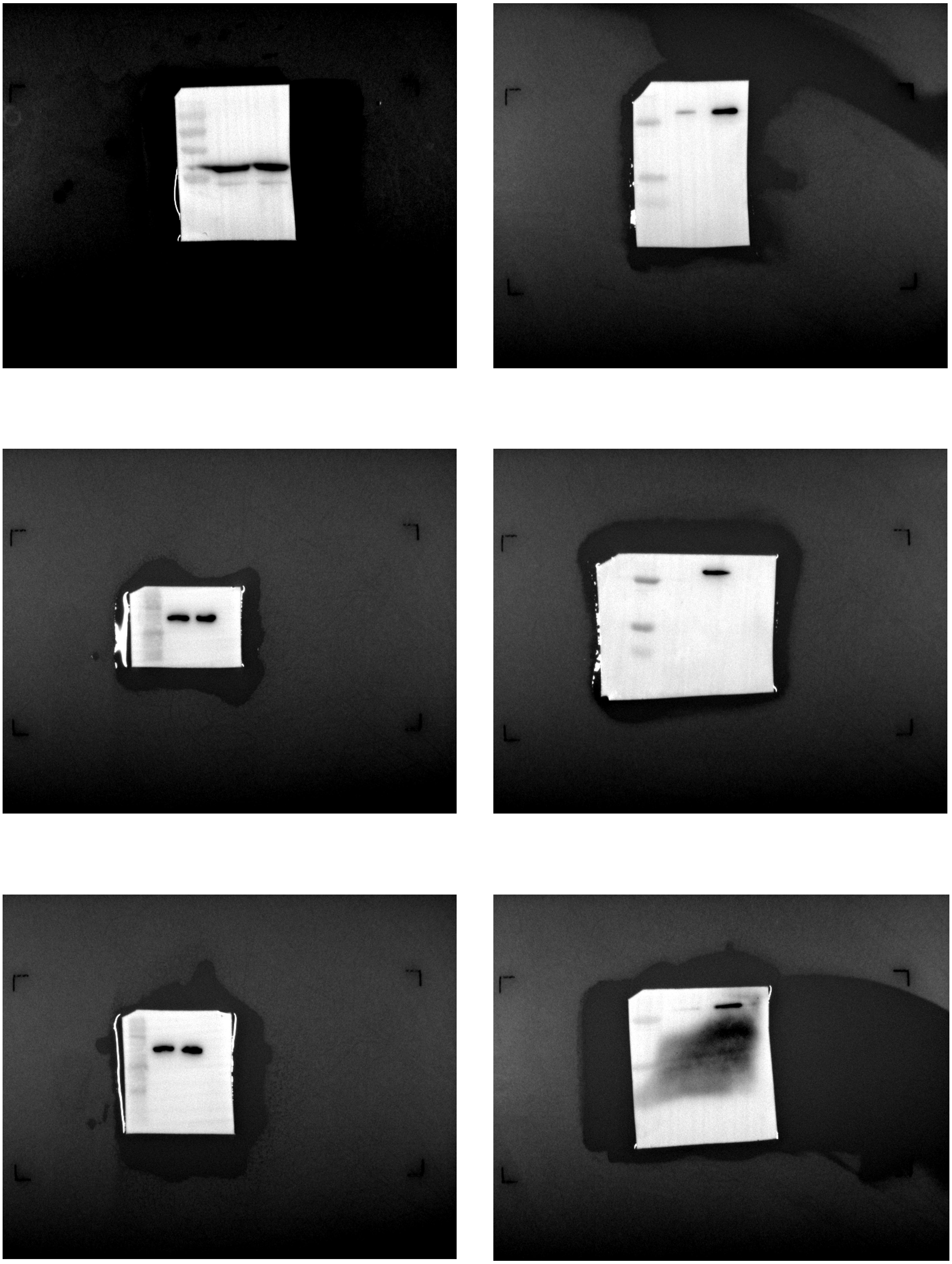

GAPDH **Noxa**

GAPDH **Noxa**

GAPDH **Noxa**

Figure 4(A) Validation of miR-200b-3p levels after transfection of AGS cells of P-Noxa group using miR-200b-3p mimic or negative control vector.

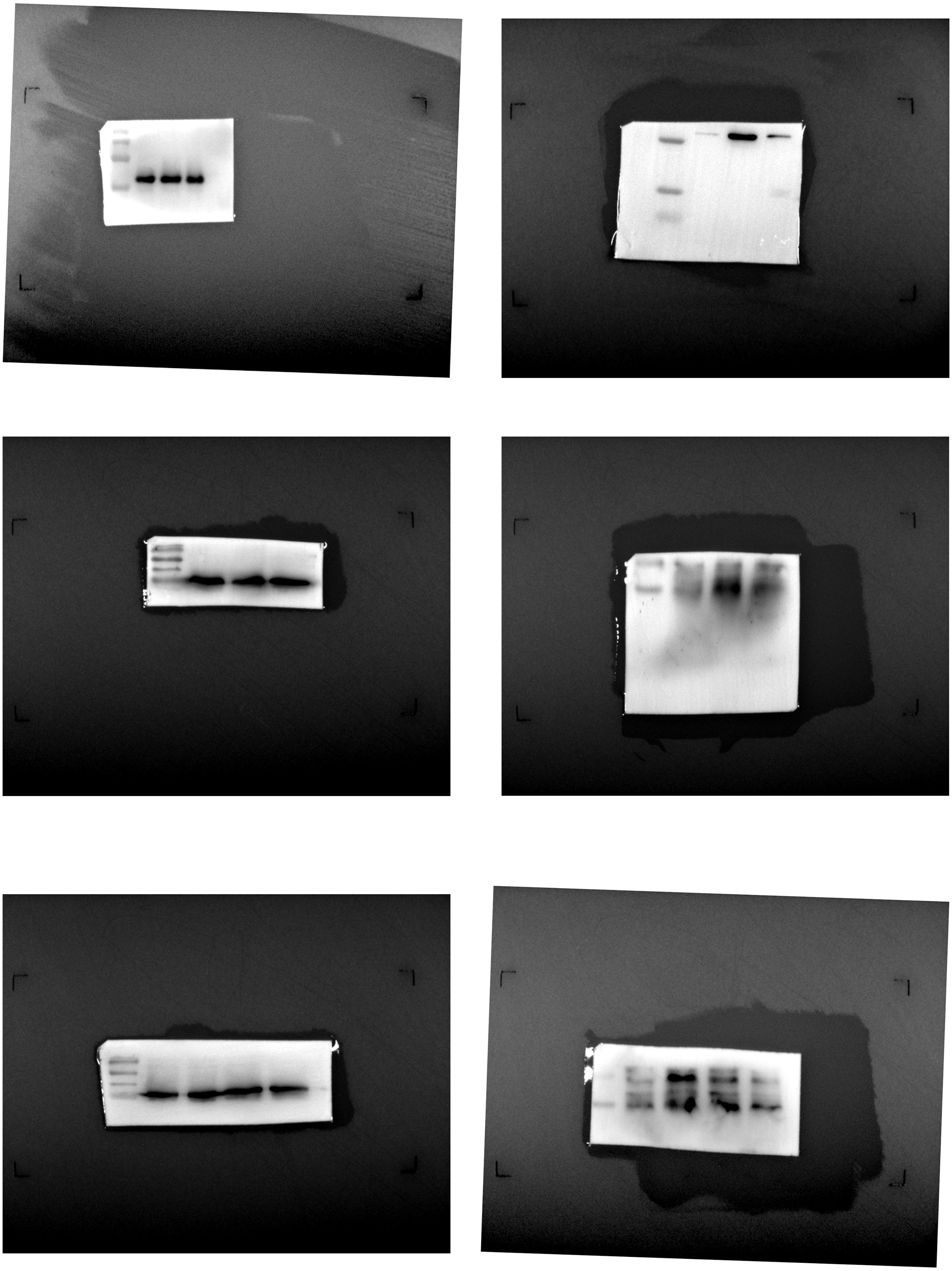

GAPDH

Noxa

GAPDH

Noxa

GAPDH

Noxa

Figure 4(A) Validation of miR-200b-3p levels after transfection of MKN-28 cells of P-Noxa group using miR-200b-3p mimic or negative control vector.

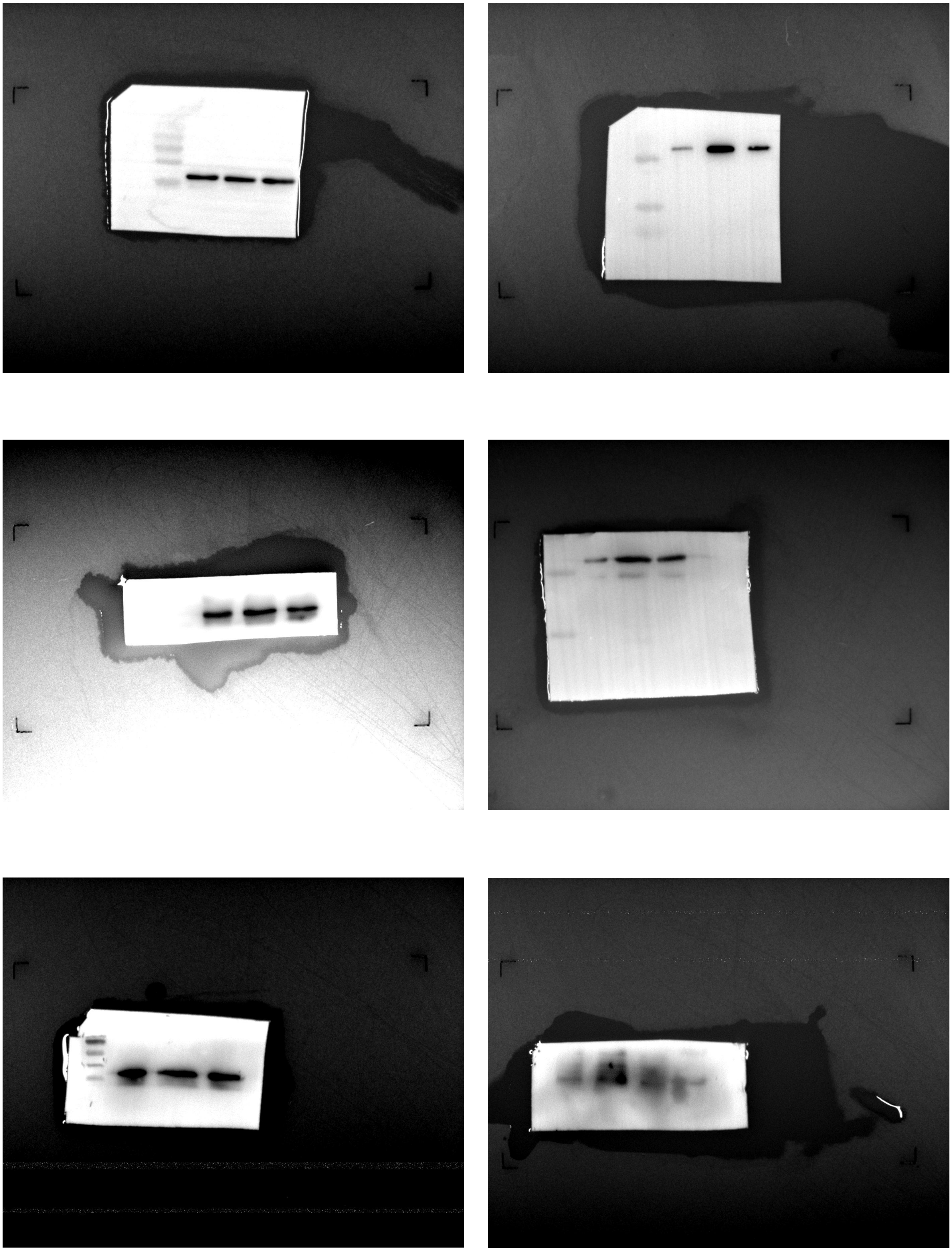

GAPDH **Noxa**

GAPDH **Noxa**

GAPDH **Noxa**

Figure 5(G) Validation of the protein levels of the downstream differential gene, ZNF519

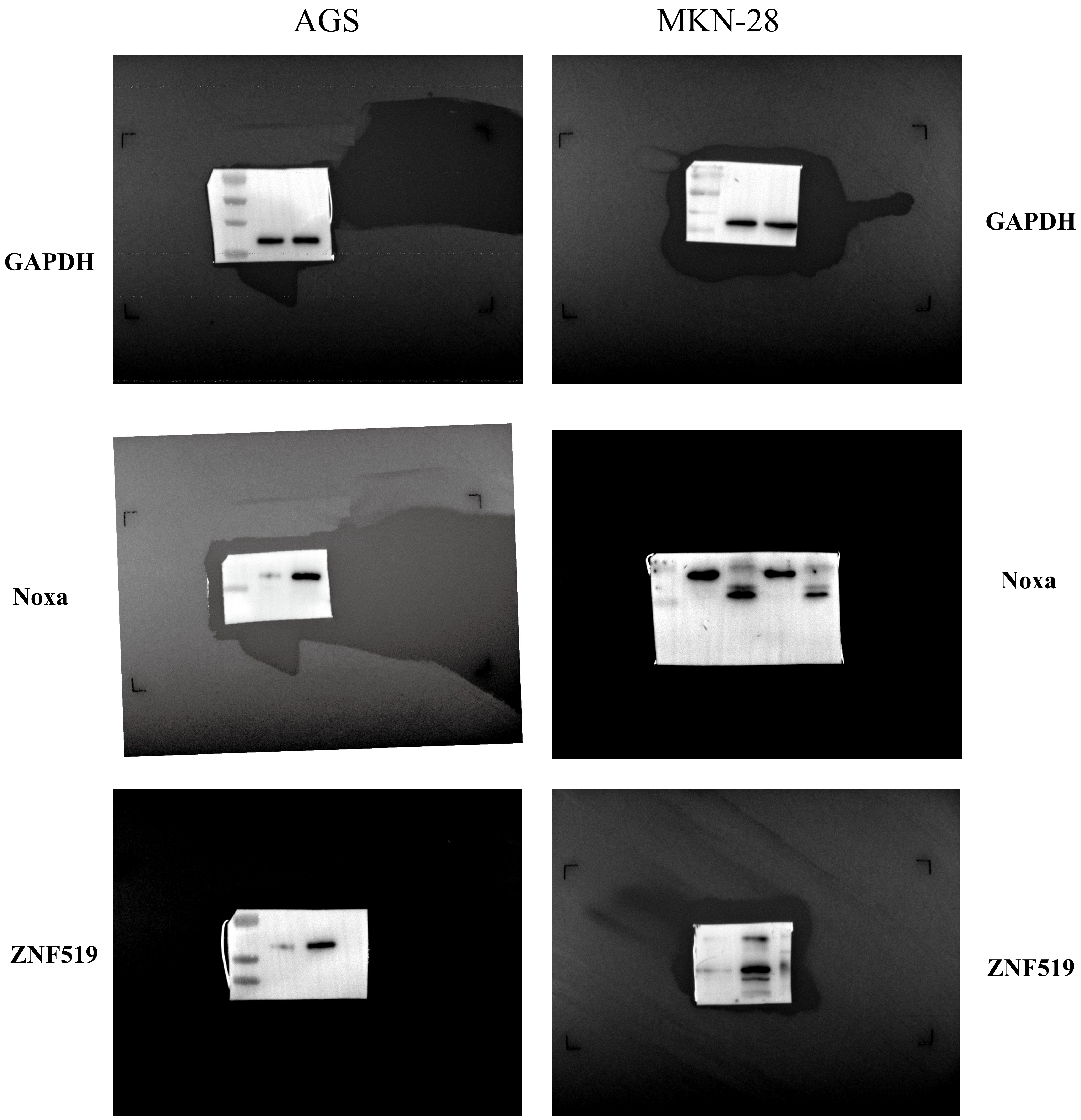

Figure 6(A-B) Validation of efficiency following the transfection of AGS and MKN-28 cells with ZNF519 siRNA

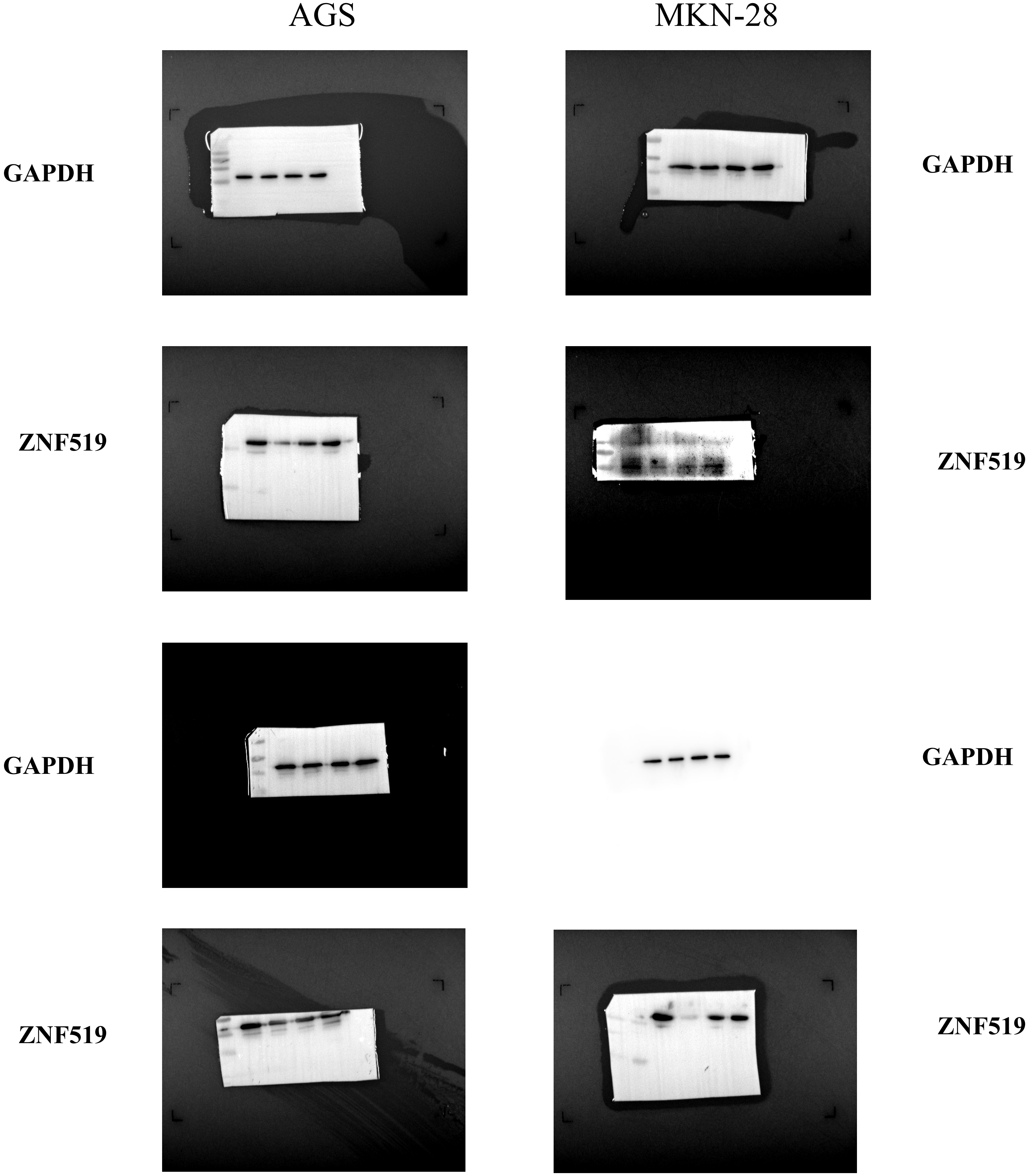

Figure 6(A-B) Validation of efficiency following the transfection of AGS and MKN-28 cells with ZNF519 siRNA

AGS MKN-28

GAPDH

ZNF519

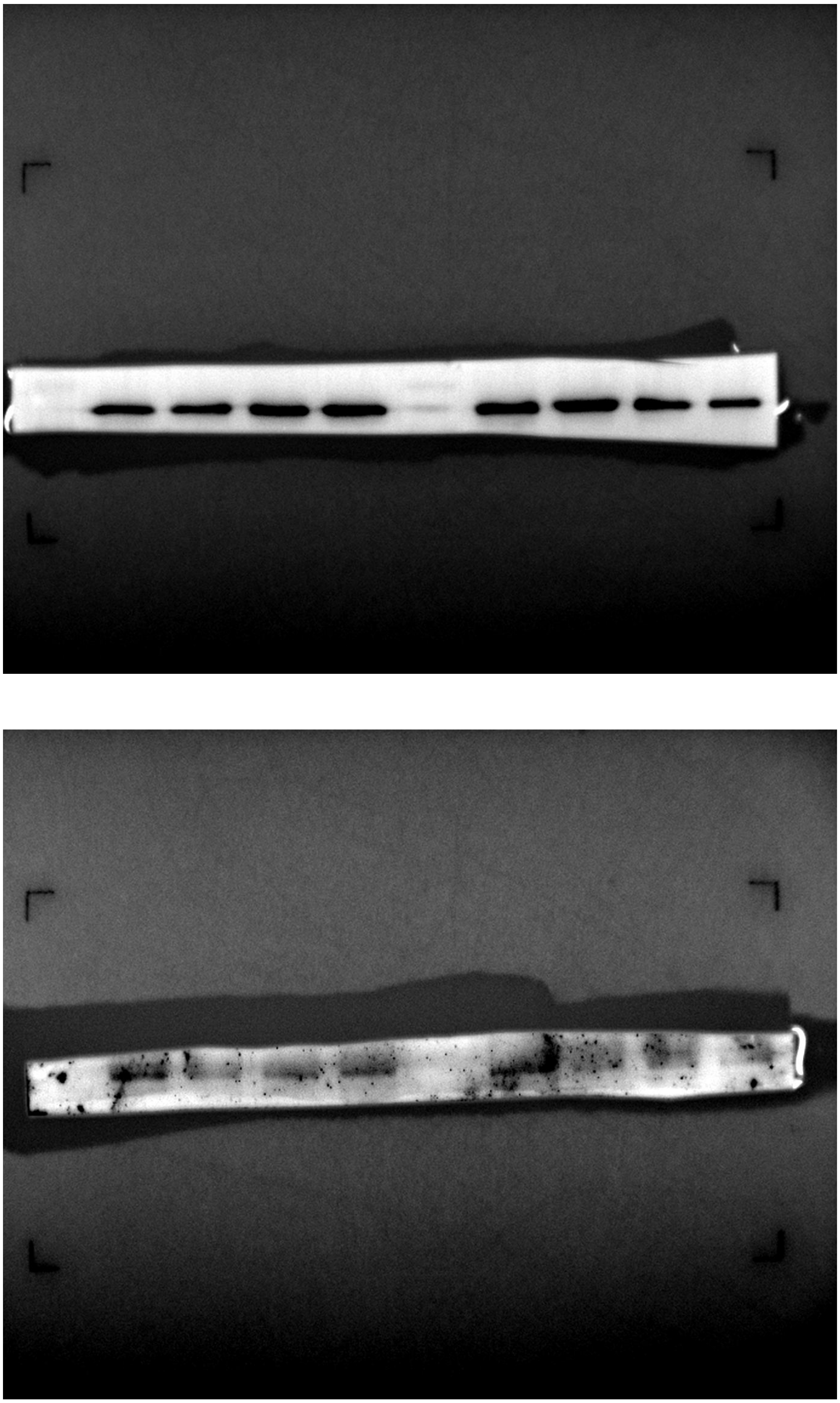

Figure 7(C) Western blot was performed to determine the protein level of ZNF519 in AGS cells transfected with vector NC, miR-200b-3p mimic, or miR-200b-3p mimic + P-Noxa.

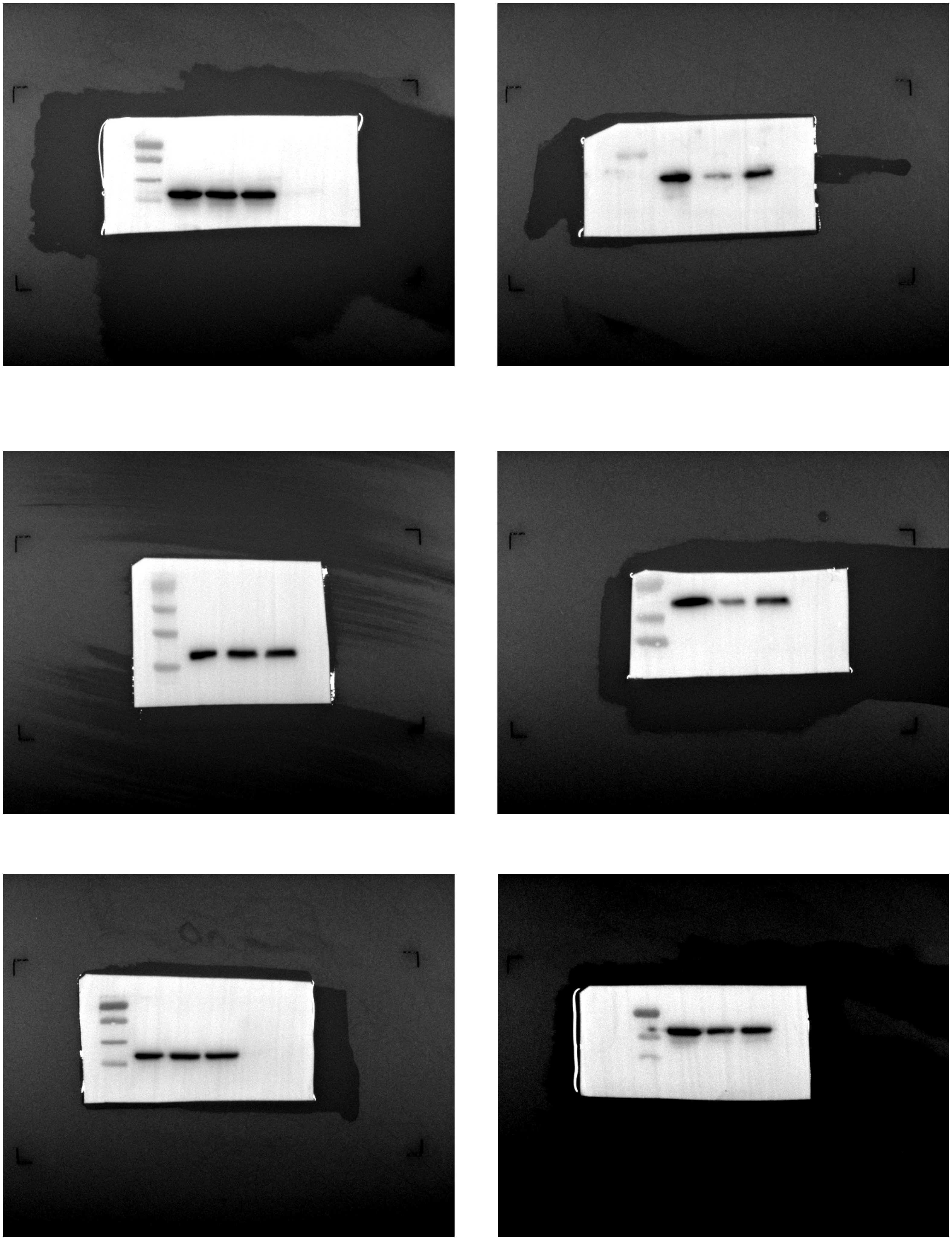

Figure 7 (D) Western blot analysis was performed in MKN-28 cells transfected with vector NC, anti-miR-200b-3p, or anti-miR-200b-3p + si-ZNF519#1 to evaluate the protein level of ZNF519.

GAPDH

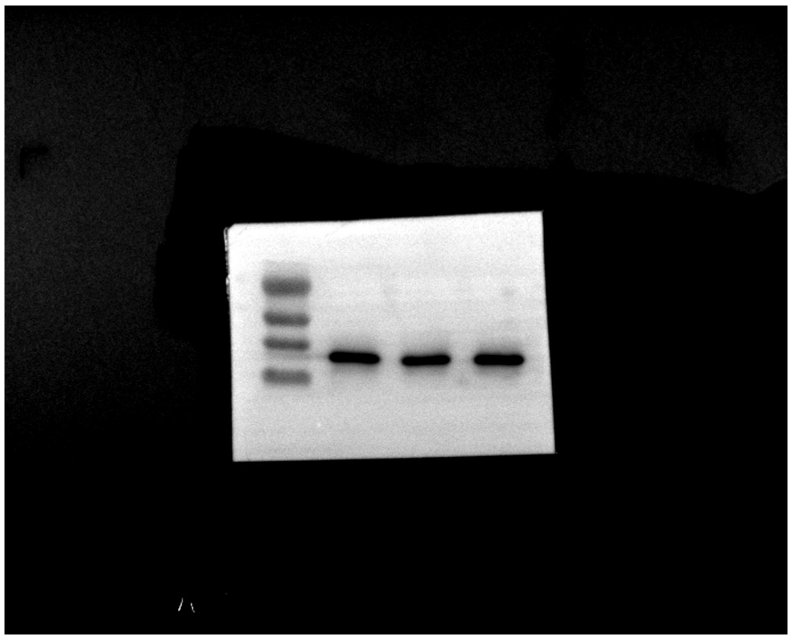

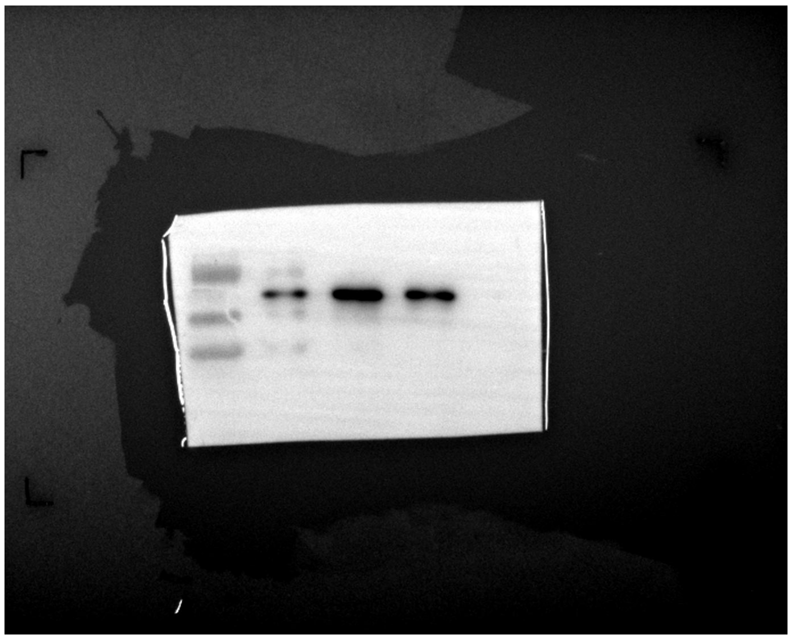

ZNF519

GAPDH

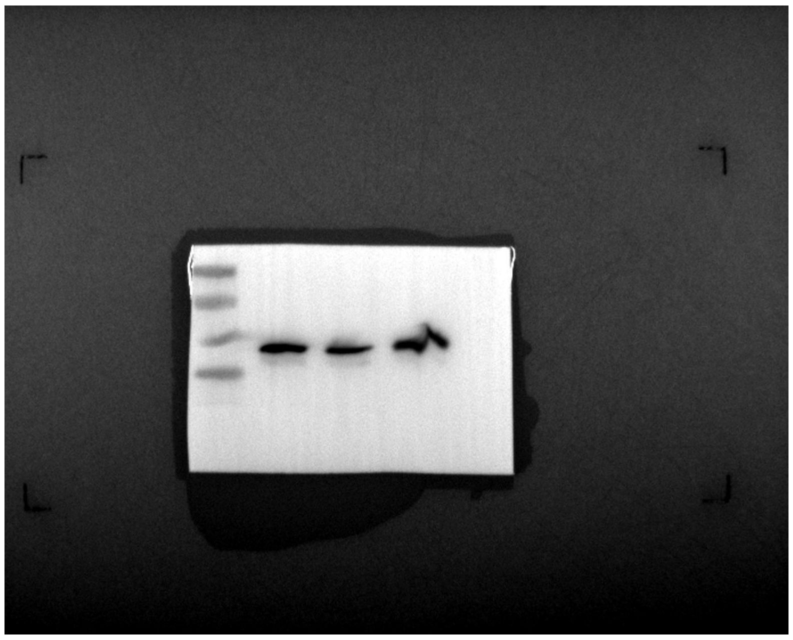

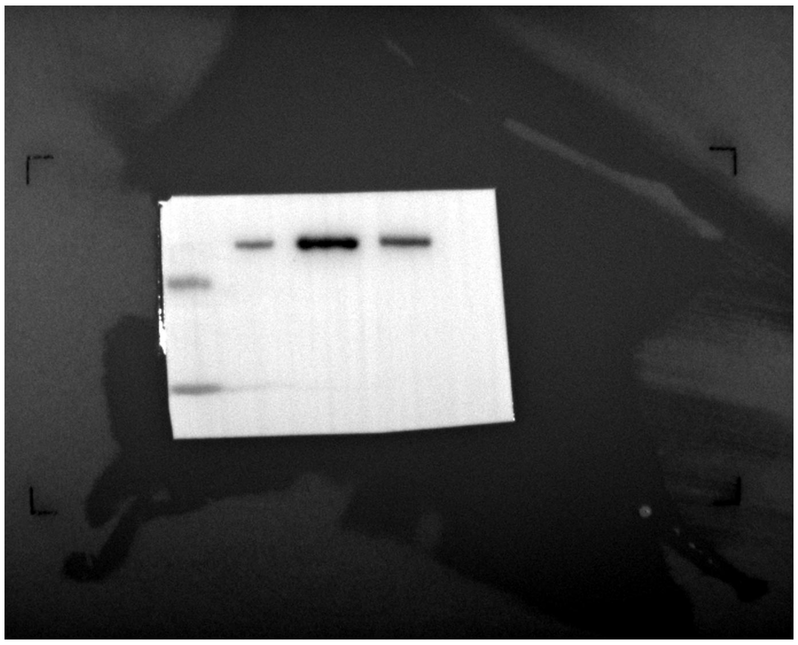

ZNF519

GAPDH

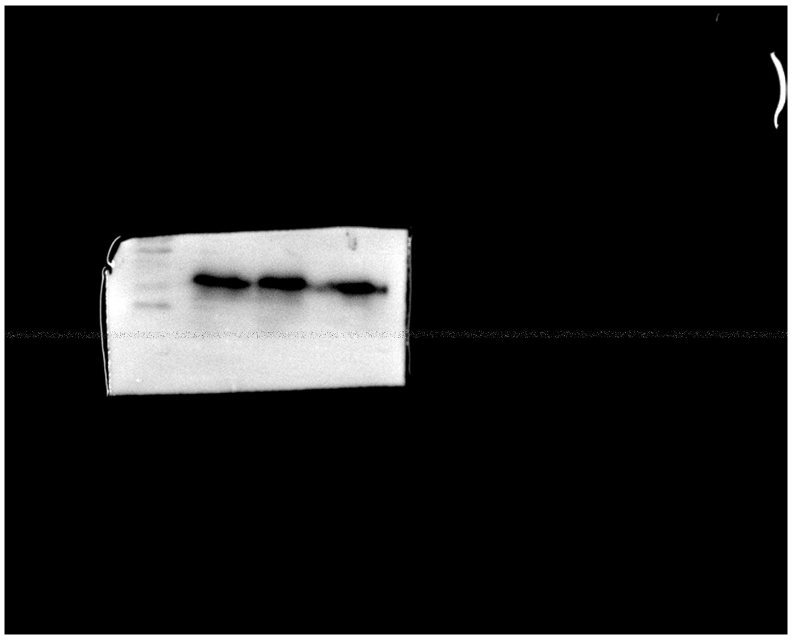

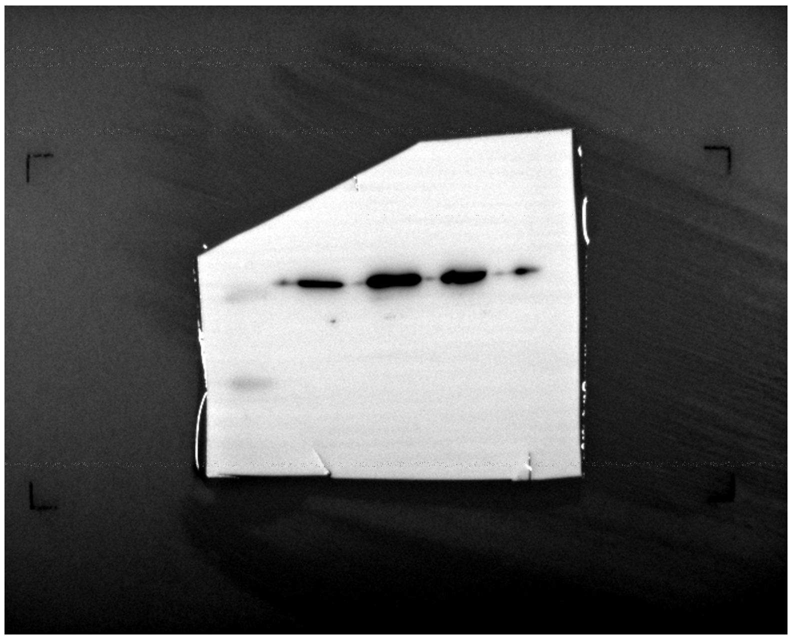

ZNF519

Figure S2(A) Validation of Noxa expression after dealing with siRNAs in AGS and MKN-28 cells through Western blot analysis.

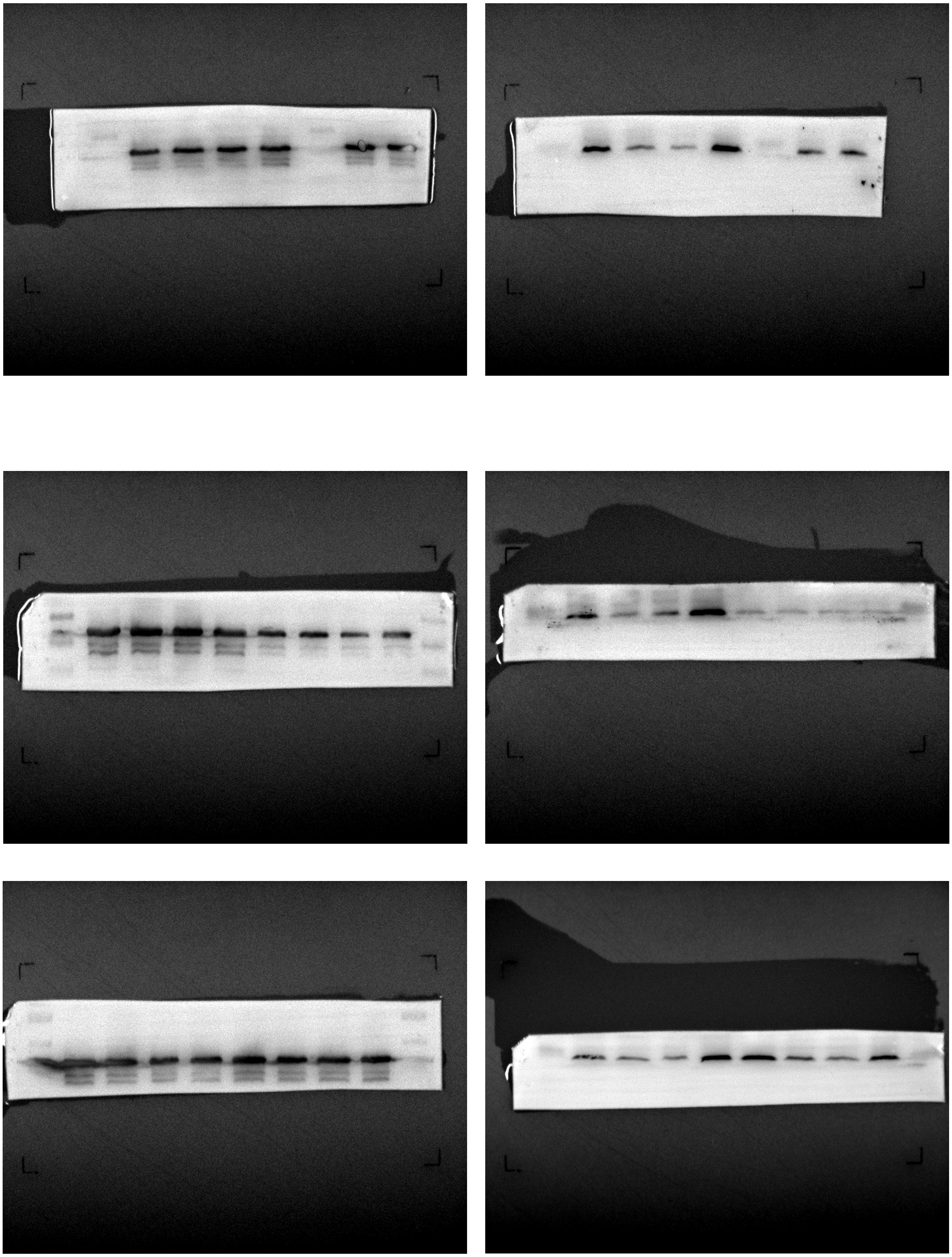

GAPDH ZNF519

GAPDH ZNF519

GAPDH ZNF519
