# Supplementary material for: Noxa inhibits oncogenesis through ZNF519 in gastric cancer and is suppressed by hsa-miR-200b-3p
Source: Sci Rep. 2024 Mar 19;14:6568. doi: 10.1038/s41598-024-57099-7 (PMC10951337; doi:10.1038/s41598-024-57099-7)
Supplement: Supplementary file 8 — Supplementary Legends. [file 41598_2024_57099_MOESM8_ESM.docx]

Figure S2: Additional Noxa expression detection; Figure S2: Additional functional assays of Noxa in vitro; Table S1: Sequences of all the shRNAs and miRNA; Table S2: Primer sequences for RT-qPCR.
